# Web-based assessment of dual-task costs at different ages: an analysis across cognitive domains

**DOI:** 10.3389/fpsyg.2025.1561417

**Published:** 2025-05-14

**Authors:** Vincenzo Livoti, Fiorella Del Popolo Cristaldi, Giulio Contemori, Maria Silvia Saccani, Mario Bonato

**Affiliations:** ^1^Department of General Psychology, University of Padua, Padua, Italy; ^2^Padova Neuroscience Center (PNC), University of Padua, Padua, Italy

**Keywords:** multitasking, aging, life-span, dual-task costs, web-based assessment

## Abstract

**Introduction:**

Understanding the trajectories of cognitive aging provides important insights that might also be potentially useful for the early detection of cognitive impairments. Among many, multitasking abilities are particularly relevant in everyday life contexts across the adult lifespan.

**Methods:**

We used web-based, self-administered, dual-tasks to measure performance and dual-task costs (DTCs) at different ages, accounting for the influence of cognitive efficiency and cognitive reserve. We also tested whether DTCs were task-specific or related to general abilities by employing three dual-tasks, each focused on different cognitive functions. We measured the performance of ﻿419 Italian-speaking healthy participants (18–76 years old) in: (i) a digital version of the Trail Making Test (A + B); (ii) the divided-attention subtest of the Test of Attentional Performance battery, adapted for online administration; (iii) a visuo-mnestic dual-task, validated in previous studies with healthy younger and older adults.

**Results:**

Results showed that with increasing age and cognitive load performance significantly reduced across all tasks. DTC for TMT and MEMO showed a small yet non-linear age-related increase. Global cognitive functioning and cognitive reserve demonstrated a weak, negative association with DTCs across all tasks, suggesting a secondary role in mediating multitasking performance. DTCs correlations across tasks were very weak, supporting the hypothesis of task-specificity for multitasking abilities.

**Discussion:**

These findings highlight the feasibility of web-based testing while also emphasizing the heterogeneity ﻿of both age-related cognitive change and the cognitive processes involving dual-task performance.﻿﻿﻿

## 1 Introduction

Cognitive abilities undergo continuous transformations across the lifespan, shaped by an interplay of biological, environmental, and experiential factors. While aging is often associated with the idea of a progressive, neural as well as cognitive decline (von Bartheld, 2018), evidence suggests that this view is simplistic if not wrong. Different cognitive functions follow distinct trajectories, with some remaining stable or even improving until late adulthood. In the context of the well-documented modifications in cognitive functioning associated with aging (Bishop et al., 2010; Murman, 2015), physiological cognitive decline does not represent a uniform process (see Brito et al., 2023 for a comprehensive review). Cognitive abilities evolve across the lifespan, with verbal skills remaining stable or improving until the 60s, while processing speed declines from early adulthood, and memory and reasoning weaken more noticeably after the age of 65 (Henninger et al., 2010; Kemper et al., 2010; Albinet et al., 2012; Bosnes et al., 2022; Salthouse, 2019). These trajectories are shaped by education, physical activity, social engagement, and cognitive reserve (Hertzog et al., 2008; Stern, 2009; Reynolds et al., 2022), making them critical factors for understanding cognitive functioning over time. Longitudinal studies showed that cognitive trajectories can help in predicting neurodegenerative risks (Liampas et al., 2024), reinforcing the urge for early diagnosis through sensitive tools to enable timely interventions (He et al., 2023).

In this study, we specifically examined from a lifespan perspective three key cognitive domains—executive functions, attention, and memory. To do so we used multitasking /dual-task paradigms, namely a promising approach for assessing cognitive functioning, particularly in the context of healthy aging or subclinical stages (Contemori et al., 2022, 2024; Saccani et al., 2022). These domains are crucial for everyday functioning and follow distinct aging trajectories, making them ideal for understanding lifespan cognitive changes. Multitasking leads to performance decline (Koch et al., 2018). Such detrimental effect can be explained by the limited capacity of attentional resources and working memory (Navon and Miller, 1987; Tombu and Jolicoeur, 2003), with effects ﻿that, sometimes, tend to intensify with age (McNab et al., 2015). This vulnerability reflects the interplay between control processes and storage mechanisms, whereby individuals with higher working memory capacity are better at resisting interference (Lorenc et al., 2021), and filtering efficiency plays a crucial role in mitigating distractor-related decline, particularly in older adults (Geiter et al., 2024). Dual-task paradigms belong to two main categories: motor-cognitive dual-tasks (MCDT) and cognitive-cognitive dual-tasks (CCDT), each with specific implications for assessing cognitive decline (McIsaac et al., 2015). In their seminal work, Lundin-Olsson et al. (1997) found that performing MCDT, such as walking while talking, significantly impaired performance in a sample of older adults. Their study revealed that greater difficulty with MCDT was associated with an increased risk of falls among participants. This detrimental effect, known as Dual-Task Interference (DTI; Pashler, 1994), has been extensively investigated across a variety of clinical populations (Leone et al., 2015; Raffegeau et al., 2019; Hamacher et al., 2014) and different age groups within healthy individuals (Schaefer et al., 2010; Bianchini et al., 2022). Nevertheless, while MCDT-induced DTI has been widely documented, less is known regarding CCDT, in particular when it comes to its clinical potential related, for instance, to the early detection of cognitive impairment in degenerative disorders. The degree of interference, referred to as dual-task cost (DTC), for some tasks can also be relatively stable across different ages yet be influenced by individual factors like cognitive reserve and educational attainment (Contemori et al., 2022, 2024; Del Popolo Cristaldi et al., 2025).

In the present study we used a set of web-based tasks to measure multitasking performance from a lifespan perspective. The advent of web-based tools offers a significant opportunity to enhance the accuracy and efficiency of cognitive assessment, particularly in remote contexts. These web-based tools address the logistical limitations of traditional testing, ensuring a measurement reliability comparable to controlled laboratory settings (Del Popolo Cristaldi et al., 2022). They also enable the collection of detailed real-time data, such as reaction times (RTs) and response patterns, or simply present stimuli in a way which allows to uncover subtle or latent cognitive difficulties often missed by standard paper-based assessments (Bonato et al., 2010, 2013).

On top of the practical advantages of web-based tools (such as automated data collection, increased accessibility, and rapid feedback), their true potential lies in the possibility to closely mimic the complexities of real-world scenarios (Casaletto and Heaton, 2017). Digital multitasking tests can align with the increasing interaction between individuals and technology in daily contexts. As highlighted by Chen et al. (2023), the integration of these real-world features with rigorous cognitive testing has positioned digital tools as a critical resource in neuropsychological practice. The growing body of evidence supporting the use of digital cognitive assessments (e.g., Kessels, 2019; Lunardini et al., 2020; Staffaroni et al., 2020) underscores their transformative potential. To conclude, digital tests are not merely practical alternatives to traditional tests but they rather seem pivotal for advancing our understanding of cognitive multitasking, particularly in contexts requiring interaction with technology.

The aim of the present study was to explore the relationship between DTCs in CCDT and their variation across the lifespan. We examined how dual-task performance differs across different age groups, identifying potential cognitive trajectories in multitasking ability for each cognitive domain. ﻿We explored to explore whether and how DTCs increase with age, and whether their impact is influenced by cognitive reserve and general cognitive functioning. We hypothesized that, regardless of the cognitive domain, performance in dual-task would have been worse than in single-task. We also expected that increasing age would have also increased DTC, with this effect potentially mitigated by protective factors such as cognitive efficiency and cognitive reserve. With the present investigation we also tested whether DTCs are associated with specific cognitive functions, reflecting task-specific effects, or whether they stem from a more global multitasking capacity, suggesting a transversal allocation of resources across different cognitive domains. This distinction is crucial for understanding the origin of DTCs. Task-specific effects would imply that certain cognitive functions are more susceptible to interference under dual-task conditions, whereas evidence for a general allocation of resources would indicate that all cognitive domains draw from a single, shared pool of limited resources, therefore leading to uniform performance decrements during multitasking. Additionally, determining whether DTCs are modular or transversal could have practical implications for designing targeted interventions, such as training programs tailored to specific cognitive skills. To achieve this, we compared DTCs across various dual-task paradigms focused on different cognitive domains, namely, memory, attention, and executive functions.

## 2 Materials and methods

### 2.1 Participants

The study initially included a total of 780 Italian-speaking participants, recruited through word of mouth and flyers distributed by the research team. Preliminary contact occurred either by phone or email, depending on participant availability. During this initial interaction, participants were provided with a general overview of the study but were kept unaware of the specific hypotheses and aims to avoid influencing their responses. Participants who expressed willingness to join were further screened based on predefined exclusion criteria which were applied to ensure a cognitively healthy sample. They included uncorrected visual or auditory deficits, neurological or psychiatric disorders, a history of alcohol or substance abuse, and a confirmed diagnosis of mild cognitive impairment (MCI) or dementia. The entire protocol was web-based and conducted remotely, with participants completing the experiment independently. Participants who met the inclusion criteria received an email invitation containing a brief description of the study’s purpose, eligibility criteria, and detailed instructions to have better control over the setting conditions and reduce potential variability due to environmental factors. They were instructed to complete the experiment alone in a quiet environment, preferably at their home, and to maintain a fixed distance from the screen (approximately 57 cm). Additionally, they were required to have a ruler available, which would be used to calibrate the task proportions to the screen size through a built-in screen adaptation feature. The email also specified that the experiment would last approximately 35 min and that data would be collected and analyzed anonymously for research purposes only. Participants could contact the research team for technical support or additional information at the end of the experiment. Some participants did not complete all the tasks, and additional participants were excluded during the post-test data cleaning process.

Only participants with complete data for all tasks entered the analysis (see “Data Analysis” paragraph for a detailed description of data cleaning procedure). After these adjustments, the final sample consisted of 419 participants (265 females) aged between 18 and 76 years (*M*: 41.85; SD: 15.56). The sample was stratified into six age clusters: emerging adulthood (18–27 years, *N* = 114), two clusters of young adulthood (28–37 years, *N* = 83; 38–47 years, *N* = 45), middle adulthood (48–57 years, *N* = 90), late adulthood (58–67 years, *N* = 69), and older age (68 + years, *N* = 18). The division into decade-based groups for adults was informed by prior research in the aging literature (Borella et al., 2008, 2014; Park and Bischof, 2013). Ten-year clusters allowed reflecting distinct psychosocial life stages while avoiding excessive fragmentation of the sample. This stratification accounted for transitions such as the shift from working life to retirement around age 67 in Italy (Ministry of Economy and Finance, Department of General Accounts, 2020). Similarly, the range for emerging adulthood (18–27 years) encompassed a phase of important transitions in personal and working life, distinguishing it from the more stable period of young adulthood. This approach aligned biological age with meaningful social and psychological phases of life. The dataset we used was partly overlapping with the previous studies by Contemori et al. (2022, 2024) for participants aged over 50.

All participants provided explicit informed consent via an online form before being included in the experiment. They did not receive any compensation for their participation. The study protocol was approved by the University of Padua’s Ethics Committee for Psychological Research (3,744 and 3,947).

### 2.2 Materials and procedures

The experimental protocol was developed using HTML (HyperText Markup Language), CSS (Cascading Style Sheets), and jsPsych (de Leeuw, 2015), an open-source JavaScript library specifically designed for web-based psychological experiments. To ensure precise timing of visual and auditory stimuli, jsPsych’s capabilities were extended using the “jspsych-psychophysics” plugin (Kuroki, 2021), which allows for accurate stimulus presentation timing. This methodology enables remote measurement of accuracy and RTs for audiovisual stimuli with laboratory precision (Bridges et al., 2020; Gao et al., 2020; Sauter et al., 2020). jsPsych runs entirely on the participant’s computer, avoiding the need for external software. The experimental materials were uploaded to a web server with a JATOS (Just Another Tool for Online Studies; Lange et al., 2015) instance installed. JATOS is an open-source platform for managing data and links to the experiment.

Each participant received a unique, one-time-use access link, which directed them to a specific combination of tasks. Task order was counterbalanced (12 combinations) to control for carryover effects and minimize interactions. Participants were randomly assigned to one of these combinations and completed the session in a single sitting. To take part in the study, participants needed a computer with internet access, a mouse, a keyboard, and audio speakers. The online protocol has been debugged in advance on three widely used browsers (Chrome, Firefox, and Edge) to ensure its correct execution. It included three digital dual-tasks and one digital screening test for the assessment of general cognitive functioning.

One of the digital dual-tasks was an adaptation of the Trail Making Test (TMT; Reitan and Wolfson, 1988). TMT is a widely used task-shifting test and includes two parts to be consecutively administered: TMT-A (the single-task condition) and TMT-B (the task-switching condition). TMT-A is a visual search and motor-speed task while TMT-B can be considered as a cognitive flexibility task (Crowe, 1998; Arbuthnott and Frank, 2000; Kortte et al., 2002). Several recent studies developed different digital versions of TMT, mostly with touch screen (Dahmen et al., 2017; Lin et al., 2021; Simfukwe et al., 2022) or mouse-and eye-tracking (Linari et al., 2022) technology. In our computer-based version of TMT, in part A, participants were asked to sequentially click with the mouse on each of the numbers from 1 to 14 in ascending order. Conversely, in part B, participants were required to sequentially press each of the numbers from 1 to 7 and the letters from “A” to “G” in ascending order but alternating between the two sequences. Numbers in TMT-A and numbers and letters in TMT-B were displayed on the screen in a fixed, semi-random order. This means that the arrangement of the targets was designed to appear random but was identical for all participants. Prior to performing the task, both TMT-A and TMT-B included written instructions on the screen accompanied by a video demonstrating how the task had to be performed. Participants could play the video example as many times as they wanted. The maximum time allowed for its completion was 120 s, after which the task was automatically interrupted, and participants were presented with the next task. Only one error was permitted; if participants made a second error, they were moved on to the next task. The number of correct responses and time of completion were recorded. While we considered the TMT-B a dual task yet it should be noted that, strictly speaking, its B version requires task shifting. Given that this component is, at least in part, present also in the other tasks for the sake of simplicity we referred to the conditions of the digital tests requiring parallel processing as “dual tasks.”

**Figure 1:**
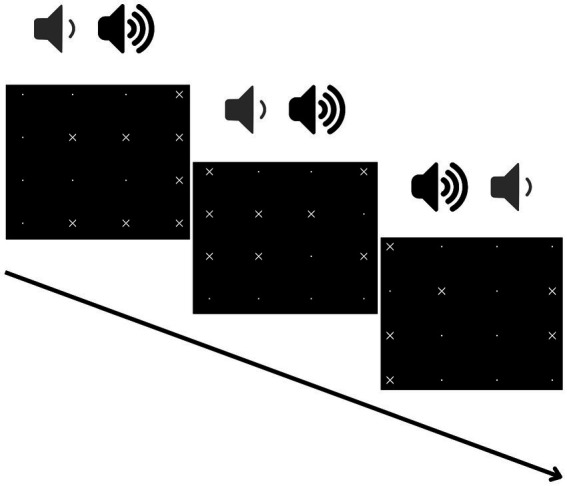
Schematic representation of the experimental paradigm for the TAP subtest in the dual-task condition. Each square represents a possible configuration of the matrix for the visual task. In the upper part gray and black volume icons represent single stimuli for the auditory task (low and high tones respectively). Specifically, the central screenshot contains the target configuration for the visual task [on the left side, four “x” forming a square, while the fifth volume icon (from the left) is a target for the auditory task (two consecutive high tones)].

Another digital dual-task included in the assessment was the Test of Attentional Performance (TAP; Zimmermann and Fimm, 2002). The TAP is a well-established computer-based battery used in the assessment of attention. Specifically, in this study we adapted its divided attention subtest. The original divided attention subtest consists in performing two simultaneous sustained attention tasks involving visual and auditory sensory modalities. The visual task required detecting a 1 × 1 square formed by four tiny “X”s randomly moving on some of the 16 intersection points of a 4 × 4 grid (Figure 1). The number of “X”s present on the screen randomly varies from six to eight. The auditory task required detecting two identical tones consecutively within a stream of alternating high and low tones. In our TAP task we separately administered the visual and the auditory tasks as two single-tasks before the combination of the two in a dual-task paradigm. Participants responded in both the single- (visual or auditory) and the dual-task conditions by pressing the “X” key only. In the visual task a total of 100 visual stimuli (17 targets) were presented, with an interstimulus interval (ISI) of 2000 ms. In the auditory task a total of 200 auditory stimuli (16 targets) each lasting 433 ms, were presented with a 1,000 ms ISI. A practice session encompassing 20% of trials was conducted prior to each experimental session.

**Figure 2:**
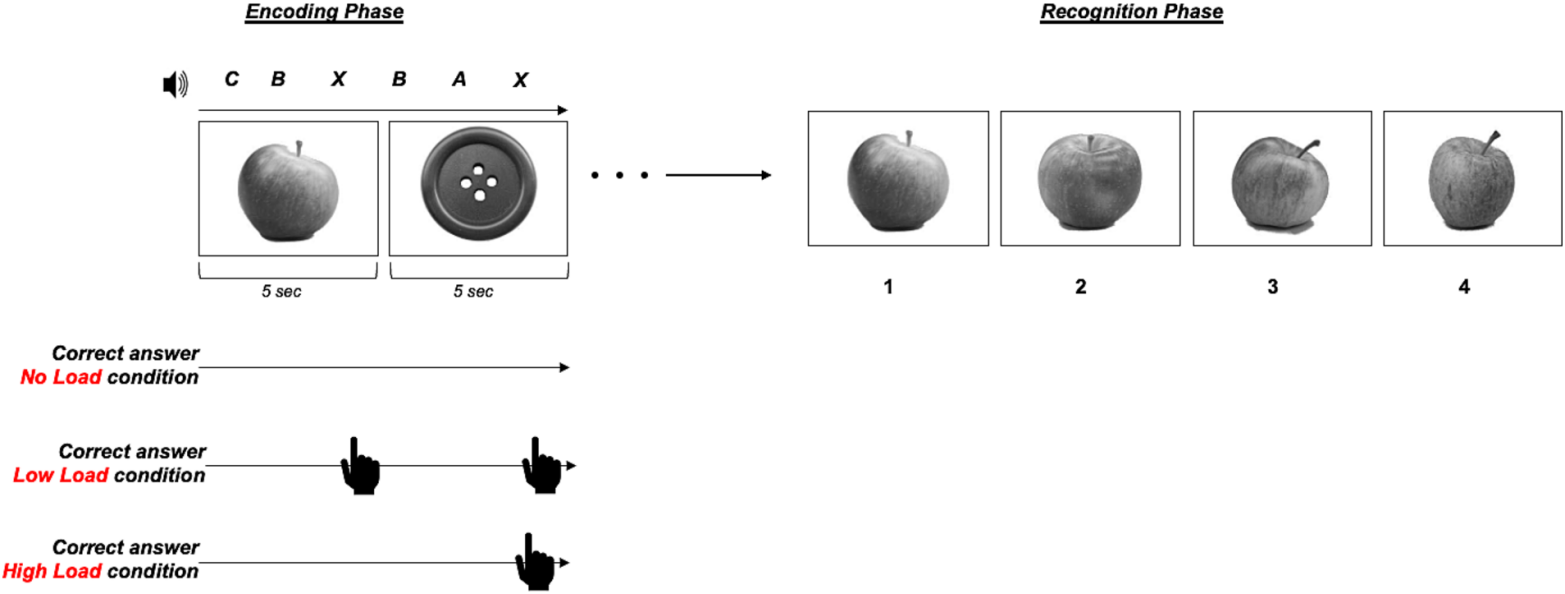
Schematic representation of the experimental paradigm for the MEMO subtest, combining a primary visual memory task and a secondary auditory sustained attention task (i.e., ACPT). The encoding phase (left) differed across conditions. In the No Load condition participants encoded the images without performing the ACPT (no response required during encoding); in the Low Load they were required to encode the images while responding to the ACPT whenever the letter “X” was presented; in the High Load condition they were required encode the images, while responding to the ACPT but only when the letter “X” was preceded immediately by the letter “A. After the encoding of the whole set of images responses were collected for each condition by using a forced-choice procedure among similar alternatives (Recognition phase, on the right).

The last dual-task paradigm we proposed will be referred to as MEMO. It consists in a digital task which has been validated in previous studies by our research group (see Contemori et al., 2022, 2024). The MEMO involved a CCDT paradigm combining a primary visual memory task and a secondary auditory sustained attention task. During the primary task (visual memory recognition) participants were required to memorize 15 sequentially presented black-and white images of inanimate objects, displayed for 5 s each. During the recognition phase, participants were presented with 4 images simultaneously (1 target image and 3 distractors of varying similarity). The position of the target among the 4 images was randomized, and participants were asked to select the image they considered the most plausible by using the number keys (1 to 4) on the keyboard. There was no time limit for the response, allowing participants to reflect before making their choice. This design emphasized familiarity-based recognition over recollection, reducing the potential influence of recollective processes on performance (Mandler, 1980; Tulving, 1985). The secondary task involved an Auditory Continuous Performance Task (ACPT, Cohen et al., 1999; Seidman et al., 2012). Participants monitored a series of auditory stimuli consisting of the letters A, B, C, D, and X. The auditory stimuli were presented with a stimulus onset asynchrony (SOA) of 1,666 ms, so that each stimulus was separated by a short interval to avoid overlap. During the image encoding phase, 3 auditory stimuli were presented during the exposure of each image, with a total of 45 auditory stimuli per session. Specifically, the MEMO paradigm (Figure 2) consisted of three experimental sessions: (i) No Load (NL): participants completed the memory task without performing the ACPT. They were instructed to ignore the auditory stimuli and focus solely on the images; (ii) Low Load (LL): during the encoding phase of the memory task, participants performed the ACPT with “X” detection, where they were required to respond whenever the letter “X” was presented; (iii) High Load (HL): during the encoding phase of the memory task participants performed the more demanding ACPT with “AX” detection, which required them to respond only when the letter “X” was preceded immediately by the letter “A..” A 2-trials practice session was conducted prior to the first experimental session. In the LL and HL conditions, participants responded by pressing the spacebar whenever the target stimulus appeared during the encoding phase.

We integrated the multitasking assessment with a measure of global cognitive functioning. Specifically, we used the Auto-GEMS (Pucci et al., 2024; see also Mondini et al., 2022 for the paper-and-pencil version and Montemurro et al., 2023 for the telephone-based version), a recently-normed web-based, self-administered test designed to provide a rapid and global measure of cognitive functioning in approximately 10 min. The test included 11 tasks that measure short-and long-term memory, visuo-spatial abilities, executive functions, spatial and temporal orientation, verbal comprehension and abstract reasoning. It provides a 0–100 score of global cognitive efficiency.

Within the Auto-GEMS protocol, 6 preliminary questions are administered before the cognitive tasks. These questions are used to automatically compute a Cognitive Reserve (CR) score (adapted from Nucci et al., 2012), a reliable predictor of overall cognitive performance. The CR score is composed of three subscores: CR-Education, which accounts for the level of formal education; CR-WorkingActivity, which reflects the type and number of years spent in various occupations; and CR-LeisureTime, which assesses the type and frequency of leisure activities. The total CR score, CR-Total, is the average of these three subscores, providing a comprehensive measure of an individual’s cognitive reserve. A value of 100 indicates the average score.

### 2.3 Data analysis

From the original sample of 780 participants we removed outliers and participants with incomplete data. Outliers were identified using the median absolute deviation (MAD) criterion (MAD > 3; Leys et al., 2019) for Accuracy and RTs in the TAP (*N* = 40) and MEMO (*N* = 33) tasks. Additional participants were excluded in both TAP (*N* = 27) and MEMO (*N* = 14) due to technical issues or unreliable answers, such as entirely missing data or the absence of responses in at least one full block of the TAP. Finally, we excluded those participants who did not complete MEMO or (*N* = 162 excluded), or TAP (*N* = 142 excluded). After this, we proceeded with TMT data cleaning. Participants who either made two errors or exceeded 120 s in TMT-A were excluded from the analysis, along with their corresponding TMT-B data (*N* = 38). The final dataset included only those participants who completed TMT-A with fewer than two errors, while TMT-B data were retained as long as TMT-A data were complete and reliable. A summary of demographic characteristics of the excluded participants is reported in Supplementary Table S1.

The data cleaning procedure above described led to a final sample of 419 participants who completed the three cognitive tasks. This allowed us to examine the effects of Cognitive Load, Age, and Cognitive Efficiency/Cognitive Reserve (CR) on task performance, including dual-task costs (DTCs). Age was always included as a six-level factor reflecting the six age clusters previously described: 18–27; 28–37; 38–47; 48–57; 58–67; 68+. The Cognitive Load factor was operationalized as the contrast of the divided attention/dual-task conditions (MEMO LL/HL, TMT-B, TAP dual) with the single-task condition (MEMO NL, TMT-A, TAP visual or auditory only).

The primary outcomes for all tasks included Accuracy, RTs, and DTC indices. For TMT we computed an Efficiency Index (EI) combining completion time, accuracy and the number of errors in a single measure calculated as reported below:


$$\mathrm{EI}=\frac{\text{Correct Responses}}{\text{Reaction Times}+\text{Number of Errors}}\times 100$$

The EIs were normalized to guarantee comparability across participants. This procedure ensures that the EIs for TMT-A and TMT-B are reported on the same scale. Accuracy and EI were coded as continuous variables ranging from 0 to 1, reflecting proportional measures of performance. RTs were transformed using a logarithmic function to address the skewed distribution commonly observed in raw RTs data, ensuring a more normalized distribution suitable for statistical analyses and improving the reliability of model estimations (see Supplementary Table S2). The DTCs for both Accuracy and RTs in MEMO and TAP, and for EI and RT for TMT were calculated as the relative difference in performance between the dual- and the single-task conditions according to the formula:


$$\text{Cost}=\frac{\text{Dual}-\text{Single}}{\text{Single}}\times 100$$

This formula captures the relative DTC performance when switching from a single-task to a dual-task condition, expressed as a percentage difference. Considering Accuracy or EI, a negative DTC indicates a decrease in performance in the dual-task with respect to the single-task. On the contrary, considering RTs the decrease in performance is indicated by a positive DTC, meaning a slowing down of responses in the dual-task condition.

The analyses were performed using (R Core Team, 2023) and had the same structure for the three experimental tasks. For all tasks, linear mixed-effects models (LMMs) were applied to analyze Accuracy/EI and RTs, with Cognitive Load, Age (as categorical variable with six levels) and their interaction as fixed effects, and random intercepts for participants. These models are well-suited for handling the hierarchical structure of the data, where repeated measures are nested within participants. This approach allows for the simultaneous modeling of both within-subject variability (e.g., changes in performance under different cognitive load conditions) and between-subject variability (e.g., differences in baseline performance across age groups). Furthermore, mixed-effects models account for the non-independence of observations within individuals and provide flexibility to include both fixed effects to estimate population-level trends and random effects to capture individual differences. This makes them particularly appropriate for analyzing complex datasets with repeated measures, ensuring robust and accurate inference even in the presence of unbalanced data. LMMs were implemented using the “lmer” function from the “lme4” package (Bates et al., 2015). DTCs related to Accuracy/EI and RTs were further examined using linear regression models to assess the influence of age, cognitive efficiency (measured by the Auto-GEMS), and Cognitive Reserve (CR) with DTC as a dependent variable and Age (as categorical variable with six levels), Auto-GEMS, and CR score as predictors. We did not include the interaction terms to avoid collinearity problems.

For the sake of brevity, results for all RTs analysis were reported in Supplementary materials only. Moreover, the inclusion of RTs in the main text was avoided because the RTs in the MEMO task were less reliable, as participants were not explicitly instructed to respond quickly. Nonetheless, the mean and standard deviation for RTs are still presented in the main text for completeness. ﻿In Supplementary materials we also reported the performance on the secondary task on MEMO (ACPT) even though our main interest was on the memory performance in the primary task (image recognition).

The significance of fixed effects and interactions was evaluated with the “Anova” function from the “car” package (Fox and Weisberg, 2019). We ran Type III ANOVA (Wald Chi-Square) for mixed models with interactions and Type II ANOVA (F-test) for linear regression with no interaction terms. Pairwise comparisons were conducted using the “emmeans” function from the “emmeans” package (Lenth, 2024) to explore differences across age clusters and conditions. The False Discovery Rate (FDR) adjustment method was used to control for Type I error inflation. For each model, we reported the Wald chi-square or F-test statistics for the predictors and interactions, as appropriate. When relevant, we provided the corresponding model fit statistics, including the regression coefficients (*β*) and *p*-values. In cases where multiple comparisons were described, we reported the estimated means, standard errors (SE), degrees of freedom (df), t-values, and p-values for each comparison. We performed partial correlation analyses using the residuals from linear models that predicted DTCs across different tasks. These models controlled for the effects of Age, Cognitive Efficiency (Auto-GEMS) and CR. By examining the correlations among residuals, we aimed to assess whether DTCs from different tasks exhibit shared variance beyond the effect of age, cognitive efficiency and CR indices. To further investigate the role of these variables in modulating the DTC, a mediation analysis was conducted for each DTC assuming Auto-GEMS or CR scores as mediating variables in the relationship between Age (coded as continuous variable ranging from 18 to 76 years old in this case) and DTCs. The analysis was implemented with two linear regression models: one predicting the mediator from age and the other predicting the DTC outcome from both age and the mediator. Mediation effects were tested using the “mediate” function from the “mediation” package (Tingley et al., 2014), with bootstrapping to estimate confidence intervals for indirect effects. The mediation analysis is reported in Supplementary materials.

## 3 Results

We examined potential sex differences in education levels (Table 1). No significant differences emerged for males vs. females within any age cluster (all *p* > 0.05).

**Table 1:** Education differences by Sex across Age Clusters.

Age cluster (years)	Education (years)		*p*-value (t-test)
	*F* (*N* = 265)	*M* (*N* = 154)	
18–27	15.2 ± 1.90	14.5 ± 1.88	0.07
28–37	16.2 ± 3.13	15.9 ± 3.03	0.64
38–47	14.6 ± 3.71	16.0 ± 2.98	0.21
48–57	13.4 ± 3.48	12.2 ± 3.19	0.12
58–67	13.2 ± 3.10	12.3 ± 3.25	0.23
68+	9.0 ± 5.04	10.3 ± 3.20	0.54

Each measure is reported as Mean ± SD. Both Age and Education are reported in years. F, female; M, male.

### 3.1 Analysis of performance

#### 3.1.1 Descriptives

Table 2 reports descriptive statistics for Accuracy/EI and RTs across all tasks and Cognitive Load conditions.

**Table 2:** Descriptive statistics for Experimental tasks.

Task	Condition		Accuracy/EI	RT (ms)
TMT	TMT-A		0.49 (0.16)	23,100 (8660)
	TMT-B		0.39 (0.15)	27,400 (9860)
TAP	Single Visual		0.94 (0.06)	930 (166)
	Single Auditory		0.99 (0.03)	647 (117)
	Dual	Visual	0.95 (0.06)*	873 (144)
		Auditory		690 (118)
MEMO Image recognition	Single		0.89 (0.13)	4,403 (2037)
	Dual Low		0.78 (0.18)	4,843 (2274)
	Dual High		0.69 (0.20)	4,976 (2510)
MEMO ACPT	Single		None	None
	Dual Low		0.97 (0.07)	863 (323.32)
	Dual High		0.97 (0.06)	754 (377.44)

Summary of accuracy/EI and RTs for TMT (a) TAP (b) and MEMO (c). All the measures are reported as: mean (SD); accuracy and EI range is 0–1; RT = reaction times; s = seconds; ms = milliseconds. *Accuracy in the dual-task condition is coded as a unique variable independently of whether the stimuli were visual or auditory while RTs were recorded separately based on whether participants responded to a visual or auditory stimulus.

#### 3.1.2 TMT

**Figure 3:**
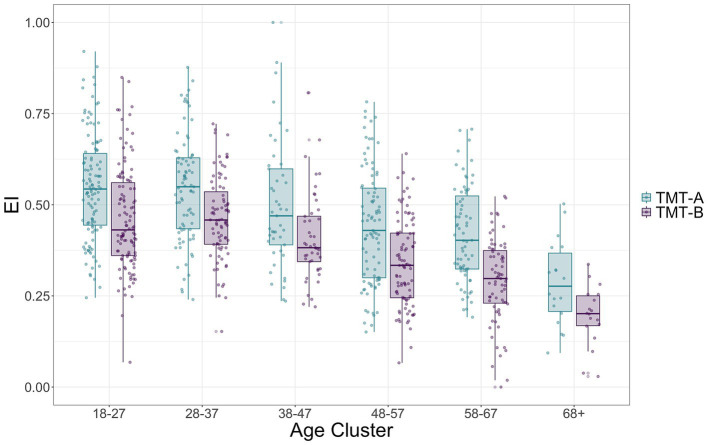
TMT-A and TMT-B EI across six Age clusters (in years). EI for TMT in both version A (cyan box-plots) and B (purple box-plots) condition is shown as a function of Age. Points show individual EI score.

ANOVA for the TMT showed a significant decrease in EI associated with increasing *Age* (Chisq(5) = 150.33, *p* < 0.001). The pattern could be better interpolated by a non-linear fit (quadratic curve, *β* = −0.057, *p* = 0.004). As expected, *Cognitive Load* significantly affected *EI* (Chisq(1) = 191.51, *p* < 0.001) with higher *EI* in TMT-A than in TMT-B (0.096, se = 0.007, df = 416, *t* = 13.771, *p* < 0.001). No significant *Age X Cognitive Load* interaction was observed (Chisq(5) = 8.49, *p* = 0.131). Results for the Wald chi-square tests for *EI* are summarized in Table 3. Age-related differences in *EI* for both TMT-A and TMT-B are shown in Figure 3. The same analysis was conducted for *RTs* (see Supplementary Table S3 and Supplementary Figure S1). In short, TMT-B was detrimental for performance, in a way that was similar for younger vs. older participants.

**Table 3:** Analysis of deviance for TMT EI as a function of Age and Cognitive Load (Type III Wald chi-square tests).

Dependent variable	Predictors	Chisq (df)	*p*-value
TMT EI	Age	150.33 (5)	**< 0.001**
	Cognitive load	191.51 (1)	**< 0.001**
	Age: Cognitive load	8.49 (5)	0.131

#### 3.1.3 TAP

**Figure 4:**
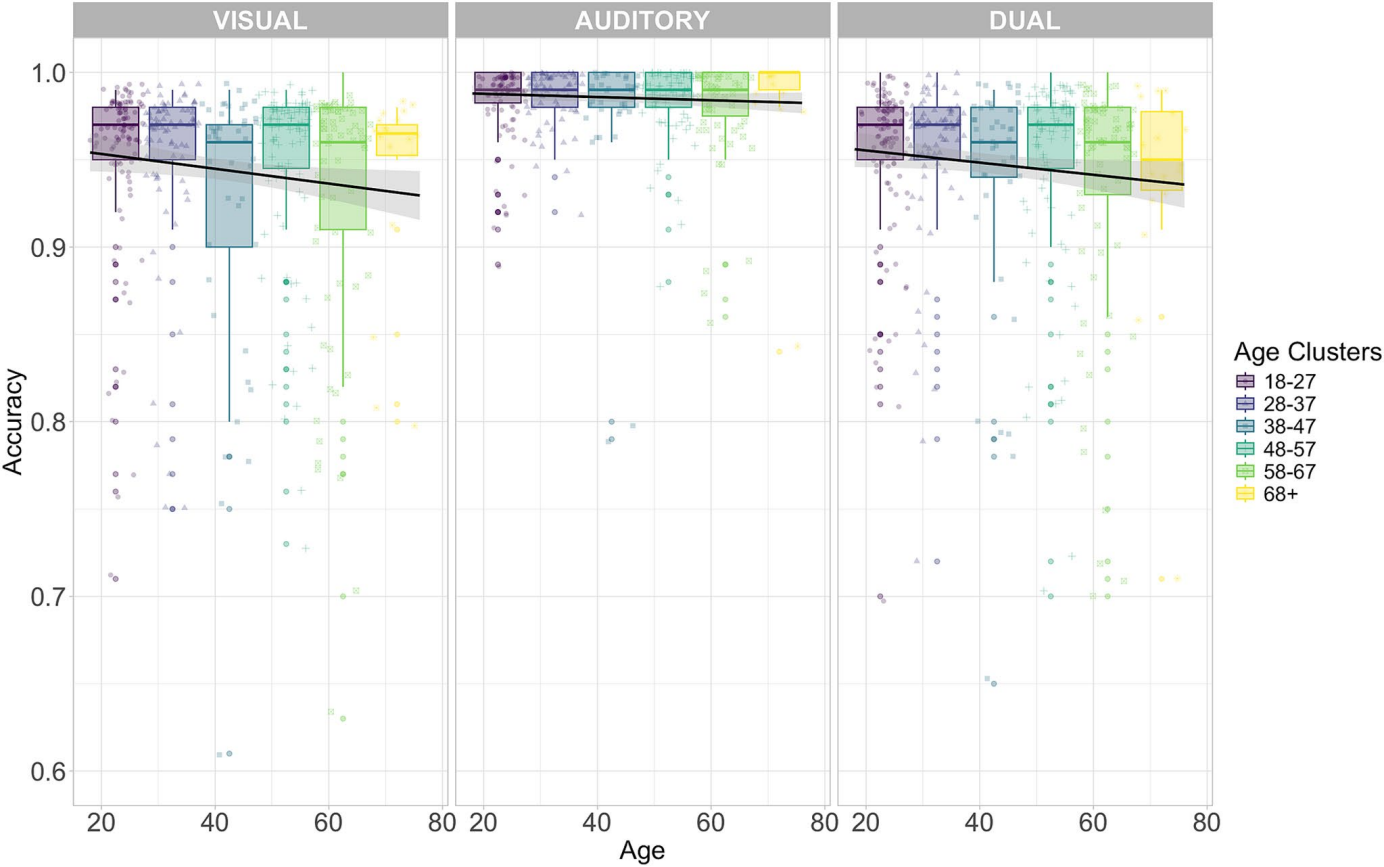
TAP Accuracy for the different Age clusters and conditions: *visual* single-task **(left panel)**, *auditory* single task **(middle panel)** and *dual*-task **(right panel)**. Each age cluster is represented by a distinct color (legend on the right). Each point represents individual accuracy. Each black line refers to the task-specific linear regression on Accuracy with Age as dependent variable. Shaded areas represent the corresponding standard errors. *Y*-axis origin is set to 0.6 instead of 0 to improve readability.

The ANOVA for the model on *Accuracy* in the TAP task showed a significant decrease due to increasing *Age* (Chisq(5) = 13.201, *p* = 0.024). Also, the modulation by *Cognitive Load* was significant (Chisq(2) = 876.165, *p* < 0.001). Performance in the dual-task was significantly worse than in the auditory single-task (0.040, se = 0.002, df = Inf, z-ratio = 24.354, *p* < 0.001). Despite a very small difference (−0.004), visual single-task Accuracy was lower than in the dual-task (se = 0.002, df = Inf, z-ratio = −2.394, *p* = 0.017). Moreover, a significant interaction between *Age and Cognitive Load* emerged (Chisq(10) = 41.299, *p* < 0.001). The 18–27 years group performed better in the auditory condition compared to the visual condition (1.12, se = 0.59, df = 120, *t* = 1.89, *p* = 0.04). In contrast, the 58–67 years group exhibited a significant decline in performance in the visual condition compared to the dual condition (1.45, se = 0.64, df = 118, *t* = 2.27, *p* = 0.02). Results for the Wald chi-square tests for *Accuracy* are summarized in Table 4. The modulation of *Age* and *Cognitive Load* on *Accuracy* is shown in Figure 4. The same analysis was conducted for RTs (see Supplementary Table S4 and Supplementary Figure S2).

**Table 4:** Analysis of deviance for TAP Accuracy as a function of Age and Cognitive Load (Type III Wald chi-square tests).

Dependent variable	Predictors	Chisq (df)	*p*-value
TAP accuracy	Age	13.201 (5)	0.024
	Cognitive load	876.1655 (2)	**< 0.001**
	Age: Cognitive load	24.354 (10)	**< 0.001**

#### 3.1.4 MEMO

**Figure 5:**
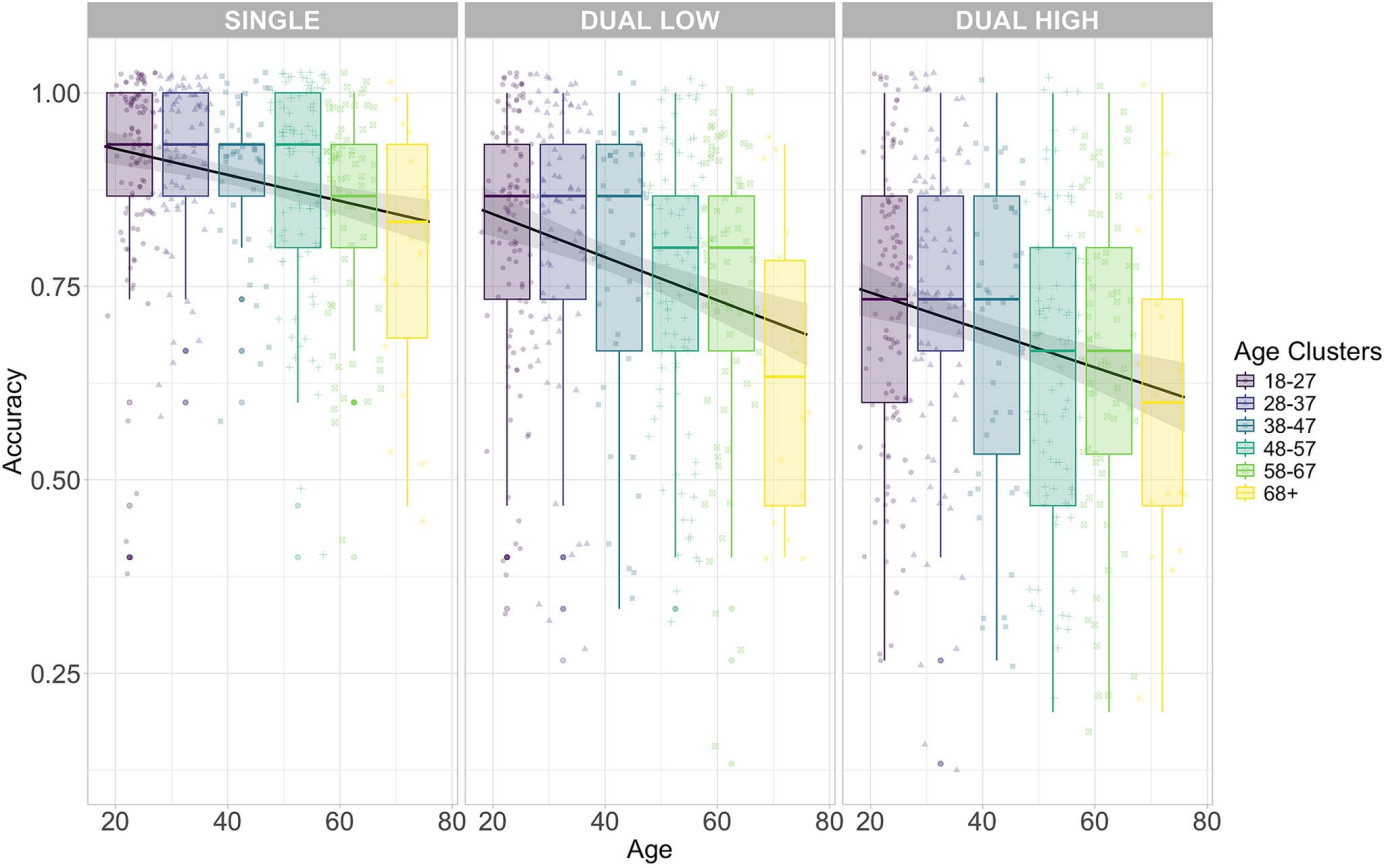
MEMO Accuracy in the primary task (image recognition) is shown as a function of Age across the three Cognitive Load conditions. Each age cluster is represented by a distinct color (legend on the right). Each point represents individual accuracy. Each black line refers to the task-specific linear regression on Accuracy with Age as dependent variable. Shaded areas represent the corresponding standard errors.

In the MEMO task, the ANOVA showed that *Accuracy* in recognizing images was significantly modulated by *Age*, (Chisq(5) = 33.76, *p* < 0.001) and *Cognitive Load,* (Chisq(2) = 1674.48, *p* < 0.001). The *Age X Cognitive Load* significant interaction (Chisq(10) = 41.33, *p* < 0.001) suggested that the detrimental effect of increased cognitive load on performance becomes more pronounced with increasing age (Figure 5). Results for the Wald Chi-square tests are summarized in Table 5. Dual-task conditions negatively affected memory performance as shown by both the NL-LL comparison (0.116, se = 0.005, df = Inf, *z*-ratio = 23.597, *p* < 0.001) and the NL-HL comparison (0.201, se = 0.005, df = Inf, *z*-ratio = 40.988, *p* < 0.001). The same analysis was conducted for RTs (see Supplementary Tables S5, S6 and Supplementary Figure S3).

**Table 5:** Analysis of deviance for MEMO Accuracy in the primary image recognition task as a function of Age and Cognitive Load (Type III Wald chi-square tests).

Dependent variable	Predictors	Chisq (df)	*p*-value
MEMO image recognition accuracy	Age	33.76 (5)	**< 0.001**
	Cognitive load	1674.48 (2)	**< 0.001**
	Age: cognitive load	41.33 (10)	**< 0.001**

### 3.2 Costs analysis

#### 3.2.1 Descriptives

Supplementary Table S7 reports DTC as a percentage calculated on Accuracy/EI and RTs for all tasks.

#### 3.2.2 TMT

**Figure 6:**
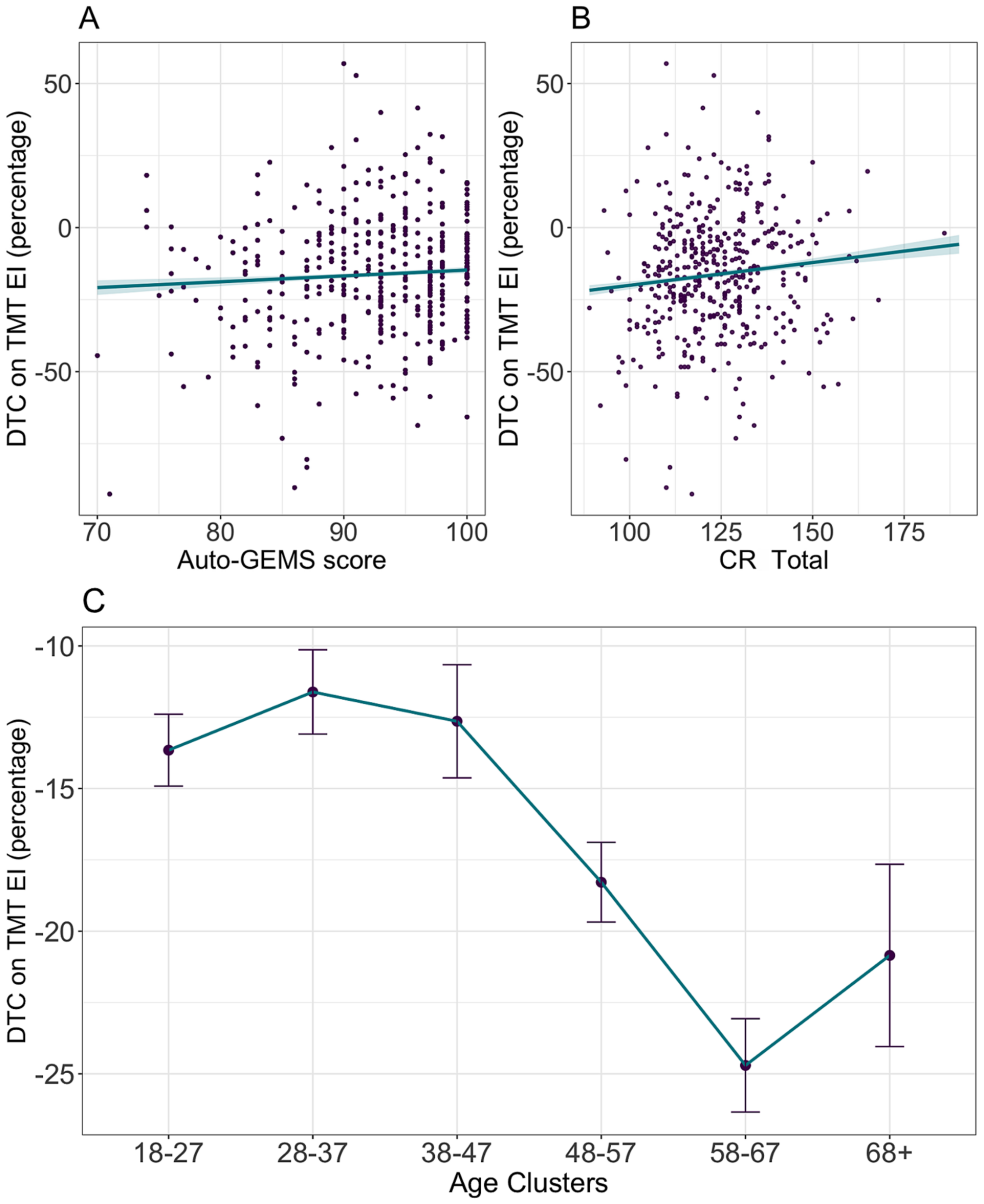
Predicted TMT DTC (percentage) on EI is shown as a function of Auto-GEMS score **(A)**, CR **(B)** and Age Clusters **(C)**. Each point **(A,B)** represents individual DTC. Shaded areas and vertical bars represent the standard error. More negative values indicate higher DTC.

The ANOVA for the model on DTC in TMT showed that *EI* was significantly influenced by *Age* (*F*(5, 3,763) = 38.335, *p* < 0.001), *Auto-GEMS*, (*F*(1, 3,763) = 16.867, *p* < 0.001) and *CR* (*F*(1, 3,763) = 34.374, *p* < 0.001). The overall pattern is reported in Figure 6 and shows a detrimental effect of Age, better interpolated by a non-linear fit (cubic curve, *β* = 6.18, p < 0.001), and a positive linear effect of *CR* (*β* = 0.14, p < 0.001) and cognitive efficiency as measured by *Auto-GEMS* (*β* = 0.22, *p* < 0.001). Results for DTC on TMT RTs are summarized in Supplementary materials.

#### 3.2.3 TAP

Type II ANOVA for the model on DTC for *Accuracy* did not show significant effects either of *Age* (*F*(5, 3,763) = 1.412, *p* = 0.217) or *CR scores* (*F*(1, 3,763) = 1.107, *p* = 0.293). *Auto-GEMS* significantly modulated *DTC on Accuracy* (*F*(1, 3,763) = 37.470, *p* < 0.001). Participants with higher cognitive efficiency showed linear (*β* = 0.08, *p* < 0.001) reduction of DTC on Accuracy (see Supplementary materials). DTC on visual and auditory RTs are summarized in Supplementary materials.

**Figure 7:**
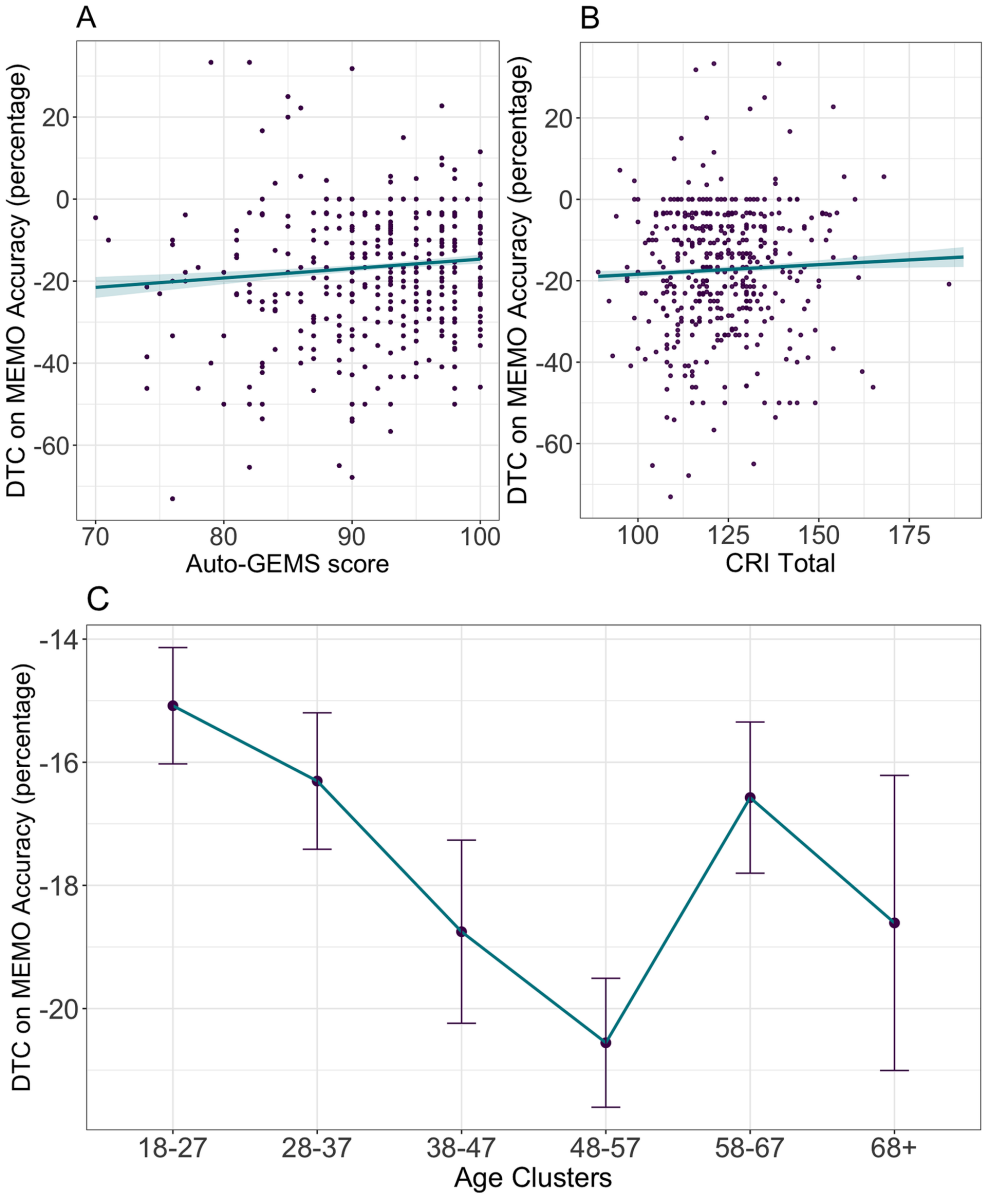
Predicted MEMO DTC (percentage) on the primary image recognition task accuracy is shown as a function of Auto-GEMS score **(A)**, CR **(B)** and Age Clusters **(C)**. Each point **(A,B)** represents individual DTC. Shaded areas and vertical bars represent the standard error. More negative values indicate higher DTC.

#### 3.2.4 MEMO

The ANOVA results for *DTC on Accuracy* model in the image recognition task revealed significant effects of *Age* (*F*(5, 3,763) = 12.93, *p* < 0.001), *Auto-GEMS* (*F*(1, 3,763) = 73.36, *p* < 0.001), and *CR* (*F*(1, 3,763) = 6.88, *p* = 0.008). The overall pattern (Figure ﻿7) shows a detrimental effect of Age, better interpolated by a non-linear fit (quadratic curve, *β* = 2.37, *p* < 0.001). Performance shows a linear increase of DTC up to the age of 38–47 years, followed by a plateau between 38–47 and 48–57 years (1.803, se = 0.928, df = 3,790, *t* = 1.944, *p* = 0.098), with a slight decrease in the subsequent age cluster (48–57 – 58-67: −3.981, se = 0.82, df = 3,790, *t* = −4.857, *p* < 0.001). The apparent increase in DTC for the oldest participants is not statistically significant (58–67 – 68+: 2.035, se = 1.367, df = 3,790, *t* = 1.488, *p* = 0.165) and is likely attributable to noise resulting from high variability within these age group. A positive linear effect of *CR* (*β* = 0.04, *p* < 0.001) and cognitive efficiency as measured by *Auto-GEMS* (*β* = 0.36, *p* < 0.001) was observed. Results for DTC on MEMO image recognition task RTs are summarized in Supplementary materials.

### 3.3 Correlation analysis: DTC on accuracy/EI between tasks

Correlations between tasks (Supplementary Figure S9) were never significant for the Memo task (MEMO–TAP: *r*_spearman_ = 0.016, *p*-value = 0.339; MEMO–TMT: r_spearman_ = −0.027, *p*-value = 0.094). The TAP - TMT correlation, albeit significant, was rather weak: *r*_spearman_ = −0.045, *p*-value = 0.006). This suggests a lack of shared variance in DTC measures across tasks controlling for Age, Auto-GEMS, and CR scores in the regression models. Each DTC measure therefore seems independent when these covariates are controlled. A similar pattern of results was observed in the correlation matrix for the raw DTC values (i.e., without controlling for covariates), which yielded comparable conclusions regarding the independence of DTC measures across tasks and the modest within-task correlations (fully reported in the Supplementary materials﻿). These findings underscore the task-specific nature of DTC measures and highlight the limited overlap in DTC variance across tasks, even when covariates are not controlled.

## 4 Discussion

In the present study we assessed the performance of 419 healthy participants, whose age ranged from early adulthood to older age, across three web-based self-administered tasks: TMT, TAP, and MEMO. These paradigms were chosen to capture core aspects of multitasking performance, including cognitive flexibility, attentional control, and memory, thereby providing a comprehensive understanding of how multitasking abilities varied across cognitive functions in the lifespan.

Concerning the overall performance, in the TMT and MEMO tasks we observed, as expected, a significant worsening of performance with increasing cognitive load. These findings confirmed the effectiveness of cognitive load manipulation in affecting task performance. ﻿In contrast, in the TAP divided attention subtest, the effect of cognitive load was less clear: while a slight performance decline was observed in the auditory condition under dual-task, no difference were found in the visual condition between single and dual task performance, suggesting that the load manipulation was only partially effective in this task. Nevertheless, these results were not totally unexpected. Only a few studies have used this subtest with healthy adults, and they mostly focused on omissions as the main outcome (Merhav et al., 2019; Schättin et al., 2019). In these studies, the number of omitted stimuli was generally low, ranging from 1 to 3 out of 33 target stimuli on average. Moreover, to the best of our knowledge, none of these studies compared dual-task conditions with visual or auditory single-task. They focused on dual-tasks mainly to explore divided attention, while we investigated other potentially relevant aspects of performance.

After having determined the effectiveness of the cognitive load manipulation we focused on how the DTC varied across the different age clusters. Our findings showed a significant increase in DTCs with age for both TMT and MEMO tasks. The dual-task condition, which is more resource-demanding than the single-task condition, becomes more challenging with increasing age. These findings align with prior research suggesting that aging disproportionately affects higher-order cognitive processes, such as cognitive flexibility, working memory and divided attention (Harada et al., 2013; Xia et al., 2024), more than basic attentional processes (Commodari and Guarnera, 2008; Madden and Langley, 2003).

Our data suggest a non-linear trend in DTC in both the TMT and MEMO task. Specifically, for TMT we detected a period of relative stability in DTC up to the 40s, followed by a linear performance decrease beyond this age. This pattern suggests that the impact of aging on DTC in the TMT task is not uniform across the lifespan. In addition, in the MEMO task DTC increases almost linearly during the early adulthood up to the 50s. Beyond the 50s, the trend reverses with a slight decrease in DTC, which eventually stabilizes, reaching a plateau between the 60s and 70s. This pattern reinforces the notion that the detrimental impact of cognitive aging is not uniform and varies depending on the cognitive domain and task demands.

Previous studies (Contemori et al., 2022, 2024; Del Popolo Cristaldi et al., 2025) focused on the effects of age, cognitive efficiency and CR in the MEMO task in a sample of healthy participants which were overall older and with a more limited age-range (50–89 years). In contrast with their initial expectations they did not find any significant increases in MEMO DTC with increasing age. Older participants within this 40-year window exhibited similar DTCs as younger participants. Here we have shown that when the sample includes young adults a significant detrimental effect of age in DTC emerges.

DTCs in the TAP task did not show any significant age-related effects suggesting less influence of aging on multitasking in this type of task. However, it is important to note that this interpretation should be considered with caution. The order of task conditions was not randomized, with visual single-tasks always preceding auditory single-tasks and then the dual-task condition. This fixed order could have led to improvements in performance due to repeated exposure, rather than reflecting actual age-related differences or cognitive load modulation effects.

In addition, we explored the role of cognitive efficiency and CR in modulating the DTC. CR resulted in a weak but significant negative relationship with DTCs in the MEMO and TMT tasks. Similarly, higher cognitive efficiency, as measured by the Auto-GEMS, was associated with reduced DTCs across all tasks. These findings, in line with previous studies (Contemori et al., 2024), underscore the need of accounting for individual differences when evaluating multitasking abilities, especially in aging (Vallesi, 2016).

Correlation analyses revealed minimal shared variance in DTC measures across the three tasks, even after controlling for Age, Cognitive Efficiency, and CR. The TMT is primarily a set-shifting task, whose B version imposes a higher cognitive load, albeit through different mechanisms than those involved in MEMO and TAP. While MEMO and TAP in the dual-task conditions require the concurrent execution of two tasks, the TMT-B demands alternating between two response sets. This difference in cognitive demands may explain the lack of strong correlations between DTCs across tasks, yet it reinforces the idea that DTCs are not merely the result of a general reduction in available cognitive resources –which also occurs in TMT-B– but rather reflect the specific nature of each task and the type of cognitive control required. All correlations were either not significant or, when significant, very low, likely due the large sample size, which increased statistical power and detected even negligible effects. The weak associations observed suggest the absence of meaningful relationships between tasks. This mirrors findings in the visual abilities research field, whereby weak correlations between performances on different visual tasks suggest limited evidence for a visual common factor. These findings were replicated even with tasks designed to measure the same visual abilities (Cappe et al., 2014; Shaqiri et al., 2019; Garobbio et al., 2024).

### 4.1 Limitations

There are a few limitations that should be acknowledged. First, the cross-sectional design does not allow for causal interpretations of the role of age and cognitive resources in DTCs. Future longitudinal studies are needed to confirm the observed non-linear patterns and to better understand how aging interacts with specific task demands over time. Second, the study was conducted using a web-based approach, which, while offering several advantages in terms of accessibility and ecological validity, introduces potential confounding factors such as variability in participants’ digital literacy and environmental conditions during self-administered assessments. These uncontrolled factors may have influenced task performance. Another potential limitation concerns the representativeness of our sample. Although we did not directly measure digital literacy, the distribution of age and education among excluded participants does not suggest that older individuals with lower education –and likely lower digital skills– were disproportionately excluded. On the contrary, the possibility of a selection bias should be acknowledged, as individuals who voluntarily participate in remote cognitive studies are expected to have at least a basic level of digital proficiency. This inherent limitation should be considered when generalizing findings to less digitally literate populations. Third, the entire protocol was conducted remotely, with participants asked to complete the experiment independently. While indirect measures were implemented to ensure compliance and minimize potential environmental influences a direct monitoring of adherence to testing conditions was not possible. Fourth, the relatively small number of participants in the older age group (68+) limits our ability to examine potential cognitive transitions occurring within late adulthood in finer detail. Nevertheless, the use of mixed-effects models in our analyses accounts for the unbalanced sample sizes across age groups, mitigating potential biases due to the lower number of participants in the older age group. Our future objectives include expanding and balancing the sample of elderly participants to better investigate the significant cognitive changes that occur in this age group. Lastly, in the TAP task, the fixed order of conditions (visual single-task, auditory single-task, then dual-task) may have led to practice effects, potentially masking the true impact of cognitive load on performance. A counterbalanced design should have been adopted to mitigate this issue.

### 4.2 Implications for research and clinical practice

In a nutshell, we have shown that performance derived from tests of different nature is reliably modulated by age and dual-task requirements. However, the weak correlations across domains strongly suggest that multitasking ability is neither a general function nor strictly linked to overall cognitive performance. We can also conclude that also the broad notion of “task difficulty” does not seem to bring together heterogeneous DTCs. While task-specific processes primarily drive multitasking efficiency, the direction and magnitude of DTCs provide critical insights into cognitive trajectories across the lifespan. Increasing age did not progressively and uniformly affect all tasks in the same way. This heterogeneity was especially pronounced in tasks requiring cognitive flexibility and memory, whereas divided attention appeared less sensitive to age-related decline.

Our comparison across different cost measures highlights the potential of web-based tools in cognitive assessment. These tools provide a sensitive and accessible means to detect subtle cognitive deficits, particularly those that may not emerge in single-task conditions. Building on their established effectiveness in assessing individual differences in memory performance (Contemori et al., 2022, 2024; Del Popolo Cristaldi et al., 2025), our results suggest their applicability across a broader range of cognitive domains. Digital assessments offer transformative possibilities for neuropsychology, enabling precise and scalable evaluations of cognitive performance. Multitasking appears particularly suited for such implementations, given its relevance in everyday life and the lack of a standardized clinical counterpart. The future development and integration of these tools into clinical workflows could enhance personalized interventions, providing a more flexible and precise approach to assessing cognitive changes across the lifespan.

Beyond its theoretical contributions, our study could have some implications for cognitive assessment and intervention strategies. Web-based dual-task assessments, such as those employed here, could serve as valuable tools for the early detection of cognitive decline, particularly in at-risk populations. To this aim, a promising avenue could be contrasting performance in those multitasking approaches resulting in age invariant-costs (Contemori et al., 2024) with performance in tasks characterized by a prominent age-related drop in performance/costs. By identifying task-specific patterns of decline, these assessments may inform targeted cognitive training programs tailored to deficits in executive function, attention, or memory. Additionally, the flexibility of online cognitive assessments makes them particularly suited for remote monitoring, allowing clinicians and researchers to track cognitive trajectories over time without requiring in-person visits. This approach could be especially beneficial for aging populations, individuals with mobility limitations, or those in geographically underserved areas. Integrating such tools into clinical workflows could optimize cognitive health management, offering a more adaptive and responsive framework for personalized interventions across the lifespan.

## Data Availability

The datasets presented in this study can be found in online repositories. The names of the repository/repositories and accession number(s) can be found at: https://osf.io/e5gy8/?view_only=caa298d059214257af699c2989c7bf26.
