# Supplementary material for: Web-based assessment of dual-task costs at different ages: an analysis across cognitive domains
Source: Front Psychol. 2025 May 14;16:1561417. doi: 10.3389/fpsyg.2025.1561417 (PMC12116685; doi:10.3389/fpsyg.2025.1561417)
Supplement: Supplementary file 1 [file Supplementary_file_1.docx]

***Supplementary Materials***

**1. Demographic characteristics of excluded participants.**

**Supplementary Table S1.** Age and Education of the excluded (N = 361) participants divided by Sex.

| **Demographics** | **Sex** | |
| --- | --- | --- |
|  | **F (N = 227)** | **M (N = 134)** |
| **Age** | 52.4 (13.6) [21–89] | 53.5 (14.1) [20–82] |
| **Education** | 13.9 (3.68) [5–21] | 13.0 (3.46) [5–19] |

**Note.** Each measure is reported as Mean (SD) [range]. Both Age and Education are reported in years. Abbreviations: F = Female; M = Male.

**2. Analysis of Performance**

Table S2 summarizes the key descriptive statistics for reaction time (RT) measures across different tasks. Mean RTs and standard deviations indicate variability in task performance, while skewness and kurtosis values provide insights into distribution characteristics. Notably, all RT measures exhibit positive skewness, suggesting a right-tailed distribution.

**Supplementary Table S2. Descriptive Statistics of Reaction Time Measures**

| **Task** | **Min** | **Max** | **Median** | **Mean** | **Std. Dev.** | **Skewness** | **Kurtosis** |
| --- | --- | --- | --- | --- | --- | --- | --- |
| **TMT-A RTs** | 10.52 | 80.89 | 21.01 | 23.05 | 8.60 | 1.88 | 9.32 |
| **TMT-B RTs** | 5.7 | 84.66 | 25.31 | 27.66 | 10.84 | 1.97 | 9.02 |
| **MEMO Image recognition RTs** | 425.18 | 20817.25 | 4137.07 | 4737.24 | 2293.65 | 2.18 | 9.96 |
| **TAP visual Rts** | 570 | 2014.56 | 877.97 | 901.63 | 158.20 | 1.71 | 9.52 |
| **TAP auditory RTs** | 436 | 1426.5 | 651.17 | 668.61 | 119.12 | 1.21 | 6.71 |

##

## **2.1. Trail Making Test (TMT) Reaction Times (RTs)**

Type III ANOVA on the LMM for RTs (Supplementary Table 1) showed a significant increase of RTs with increasing Age (Chisq(5) = 157.96, p < .001), explained by a quadratic curve (β = .153, se = .045, df = .04, t = 3.473 p < .001). and Cognitive Load (Chisq(1) = 182.59, p < .001) whereby, as expected, on average the B version took longer to be completed than the A version (-.185, se = .0138, df = 398, t = -13.382, p < .001). The interaction between Age and Cognitive Load was also significant, (Chisq(5) = 15.65, p = .008) indexing that the impact of task difficulty differently slowed completion time depending on the specific age cluster (Supplementary Figure 1).

**Supplementary Table S3.** Analysis of deviance with the type III Wald chi-square tests for TMT RTs as a function of Age and Cognitive Load.

| **Dependent Variable** | **Predictors** | **Chisq (df)** | **p-value** |
| --- | --- | --- | --- |
| TMT  RTs | Age | 157.96 (5) | **< .001** |
|  | Cognitive Load | 182.59 (1) | **< .001** |
|  | Age:Cognitive Load | 15.65 (5) | **.008** |


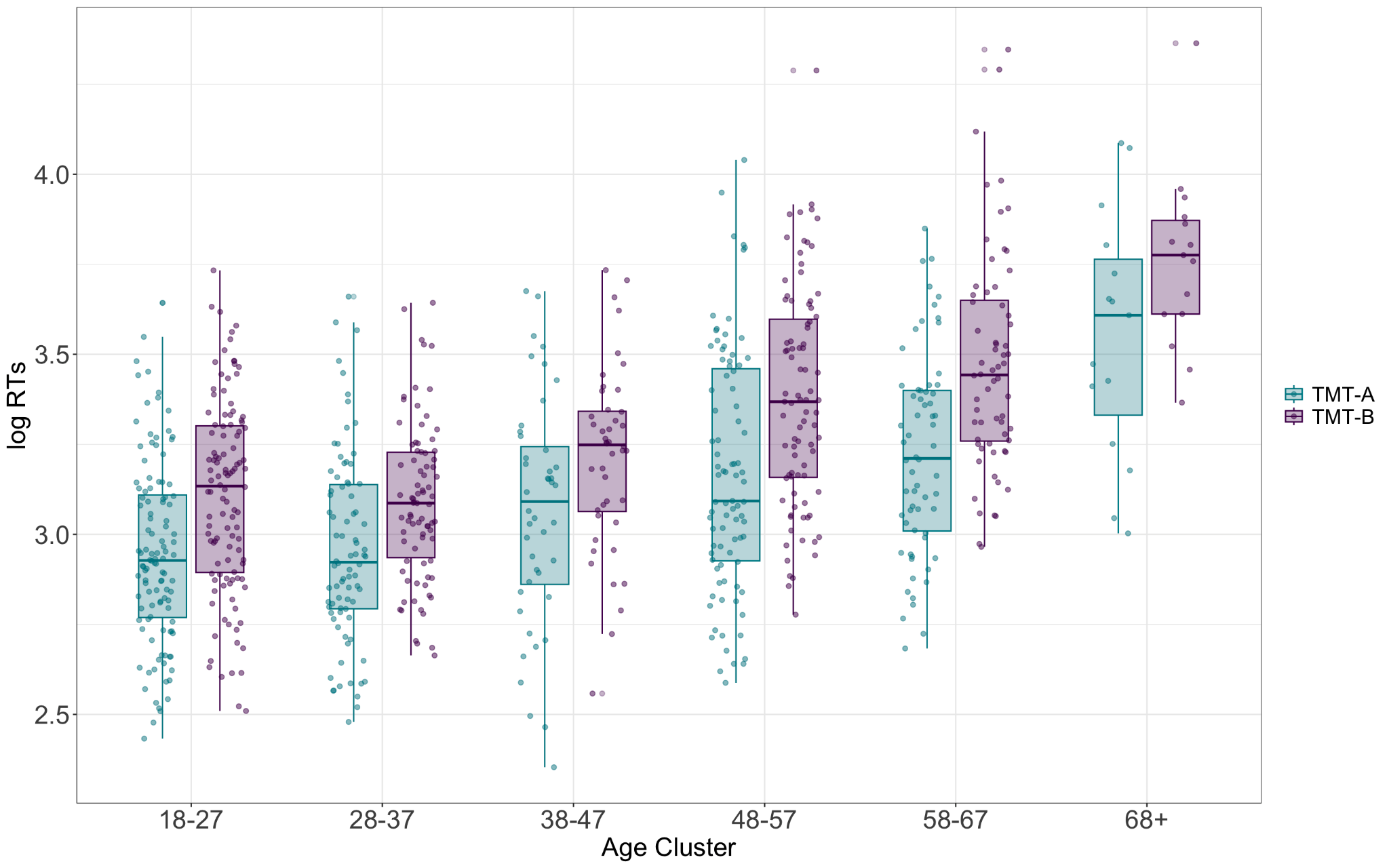


**Supplementary Figure 1.** TMT-A and TMT-B log-transformed RTs across Age. Log-transformed RTs for TMT in both A (cyan box-plots) and B (purple box-plots) condition are shown as a function of Age. Points show individual log-transformed RTs

**2.2. Test of Attentional Performance (TAP) RTs**

Type III ANOVA on the LMM for *visual RTs* (Supplementary Figure 2), significant effects were observed for Cognitive Load (Chisq(2) = 303.31, p < .001), Age (Chisq(5) = 119.10, p < .001), Age X Cognitive Load interaction, (Chisq(10) = 33.30, p < .001). A similar pattern was observed for *auditory RTs*: Cognitive Load (Chisq(2) = 323.54, p < .001), Age, (Chisq(5) = 7.49, p = .19), and the interaction, (Chisq(10) = 38.56, p < .001). Results for the Wald chi-square tests are summarized in Supplementary Table 2.

**Supplementary Table S4.** Analysis of deviance with the type III Wald chi-square tests for TAP RTs as a function of Age and Cognitive Load.

| **Dependent Variable** | **Predictors** | **Chisq (df)** | **p-value** |
| --- | --- | --- | --- |
| TAP Visual  RTs | Age | 119.10 (5) | **< .001** |
|  | Cognitive Load | 303.31 (1) | **< .001** |
|  | Age:Cognitive Load | 33.30 (5) | **< .001** |
| TAP Auditory  RTs | Age | 7.49 (5) | .19 |
|  | Cognitive Load | 323.54 (1) | **< .001** |
|  | Age:Cognitive Load | 38.56 (5) | **< .001** |


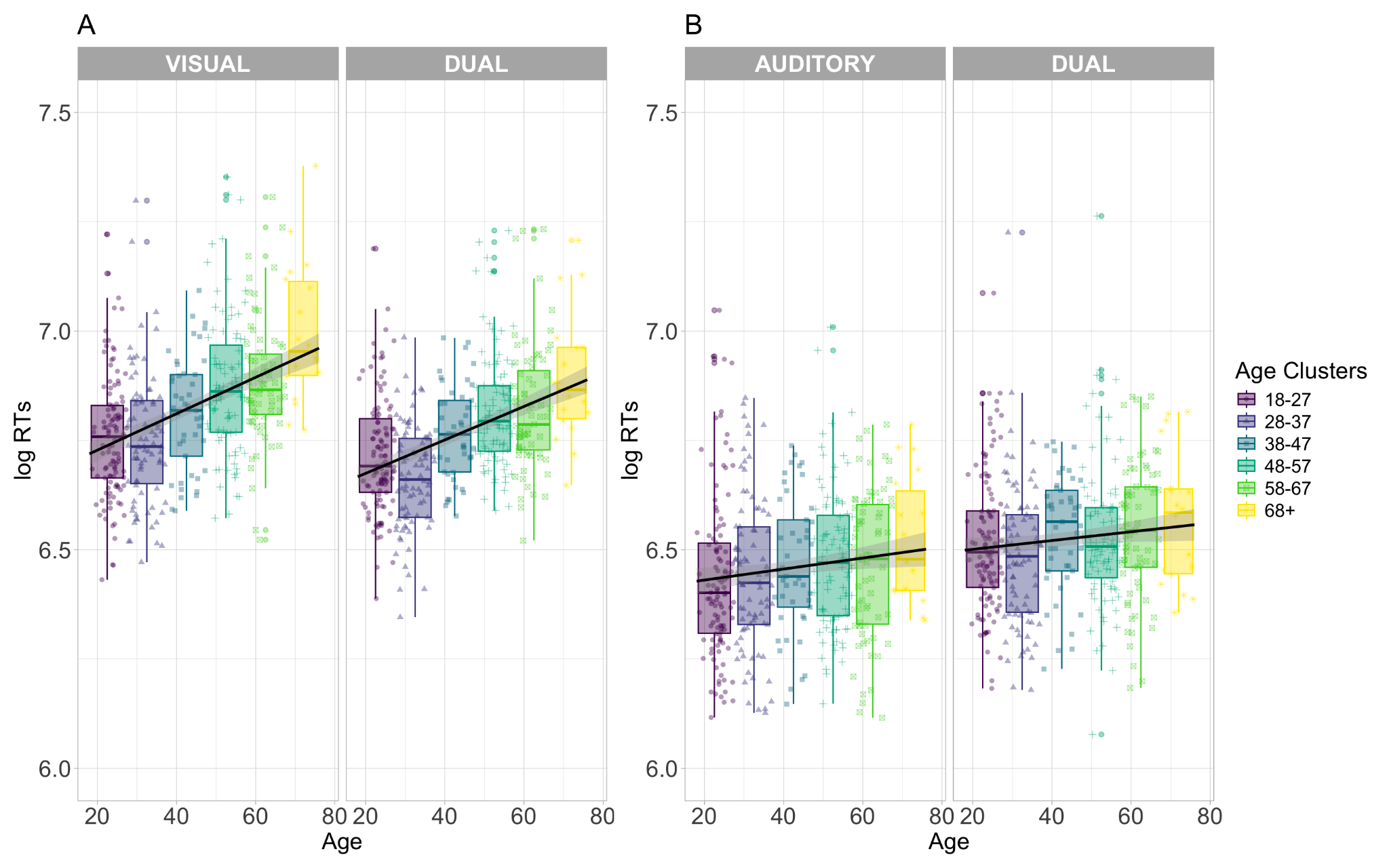


**Supplementary Figure 2.** TAP log-transformed RTs across Age and conditions. TAP log-transformed RTs are displayed for the visual task (A) and the auditory task (B) under single (left panels) and dual-task conditions (right panels). Each age cluster is represented by a distinct color, as indicated in the legend on the right. Each point represents individual log-transformed RTs. Each black line refers to the task-specific linear regression on log-transformed RTs with Age as a dependent variable. Shaded areas represent the corresponding standard errors.

**2.3. MEMO**

2.3.1. **Image recognition RTs**

Type III ANOVA on the LMM for RTs in the image recognition task (Supplementary Table 3) revealed significant main effects of Cognitive Load, (Chisq(2) = 142.81, p < .001), Age, (Chisq(5) = 103.31, p < .001), and their interaction, (Chisq(10) = 23.87 , p = .007). This suggests that RTs linearly slowed down (Supplementary Figure 3) with increasing age (β = .165 se = .048, df = 526, t = 3.451 p < .001) and with increasing cognitive load (LL - HL: .132, se = .012, df = 2035 t = 11.265 p <.001).

**Supplementary Table S5.** Analysis of deviance with the type III Wald chi-square tests for MEMO RTs in the primary image recognition task as a function of Age and Cognitive Load.

| **Dependent Variable** | **Predictors** | **Chisq (df)** | **p-value** |
| --- | --- | --- | --- |
| MEMO  Image Recognition  RTs | Age Clusters | 103.31 (5) | **< .001** |
|  | Cognitive Load | 142.81 (2) | **< .001** |
|  |  |  |  |
|  | Age:Cognitive Load | 23.87 (10) | **.007** |


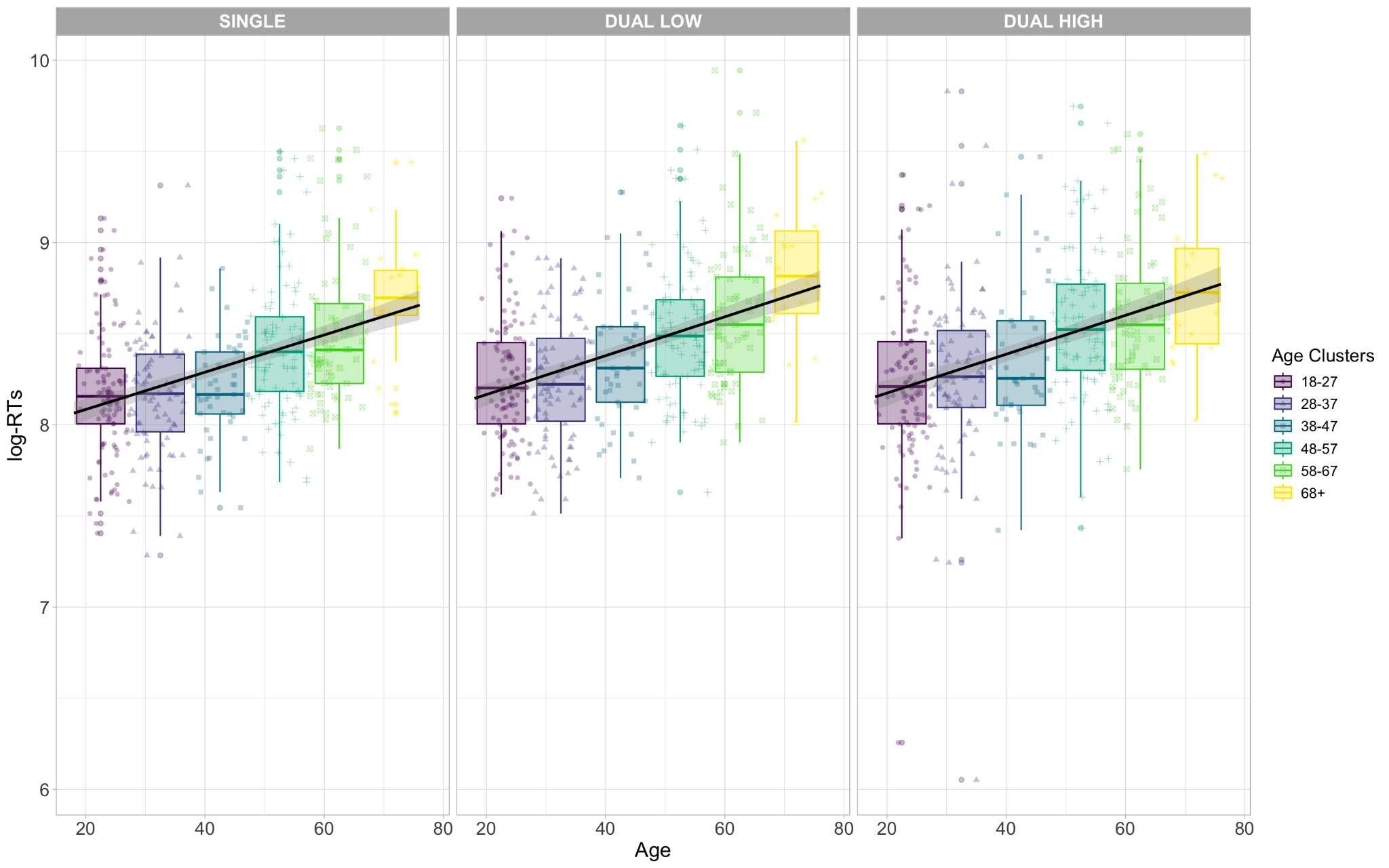


**Supplementary Figure 3.** MEMO log-transformed RTs in the primary image recognition task. Log-transformed RTs for the image recognition task are shown as a function of Age across the three Cognitive Load conditions. Each age cluster is represented by a distinct color, as indicated in the legend on the right. Each point represents individual log-transformed RTs. Each black line refers to the task-specific linear regression on log-transformed RTs with Age as a dependent variable. Shaded areas represent the corresponding standard errors.

**2.3.2. Auditory Continuous Performance Test (ACPT)**

Type III ANOVA on the LMM for Accuracy in the secondary ACPT task (Supplementary Table 4) revealed no significant main effects of Cognitive Load (Chisq(2) = 3.21, p = .07) suggesting that the performance in the secondary task was not affected by the intensity of cognitive load. A detrimental effect of Age (Chisq(5) = 27.31, p < .001) was found. The Age X Cognitive Load interaction, (Chisq(10) = 3.21, p = .007) was significant but the only difference was found for the 48-57 y.o. cluster which performed significantly better in the Low Load (LL) condition rather than in the High Load (HL) the condition (LL-HL: -.0102, se = .004, df = 2104, t = -2.825, p = .018).

**Supplementary Table S6.** Analysis of deviance with the type III Wald chi-square tests for MEMO Accuracy and RTs in the secondary ACPT task as a function of Age and Cognitive Load.

| **Dependent Variable** | **Predictors** | **Chisq (df)** | **p-value** |
| --- | --- | --- | --- |
| MEMO  ACPT  Accuracy | Age Clusters | 27.31 (5) | **< .001** |
|  | Cognitive Load | 3.21 (1) | .07 |
|  | Age:Cognitive Load | 16.52 (5) | **.006** |
| MEMO  ACPT  RTs | Age Clusters | 16.62 (5) | **.005** |
|  | Cognitive Load | 124.49 (1) | **< .001** |
|  |  |  |  |
|  | Age:Cognitive Load | 16.15 (10) | **.006** |

**3. Cost Analysis**

**Supplementary Table S7. Descriptive statistics for the DTC**

| **Task** | **DTC Accuracy/EI** | **DTC RTs** | |
| --- | --- | --- | --- |
|  |  |  |  |
| TMT | -16.31% (21.06) | 23.09% ( 33.94) | |
| TAP | -1.8% (4.7) | Visual | Auditory |
|  |  | -0.42% (1.48) | 7.68% (15.17) |
| MEMO Image recognition task | -17.3% (15.62) | 12.88% (35.56) | |

**Note:** Summary of percentage DTCs on Accuracy/EI and RTs for all tasks. All the measures are reported as: mean (SD); RTs = reaction times. For DTC Accuracy/EI, negative values represent a decrease in performance in the dual-task condition compared to the single-task condition, while positive values indicate an increase. For RTs, negative values represent an increase in performance in the dual-task condition compared to the single-task condition, while positive values indicate a decrease.

**3.1. TMT Dual Task Cost (DTC) on RTs**

Type II ANOVA on the LM for *RT* DTC showed significant effects of Age, (F(5, 3763) = 35.67, p < .001), and CRI, (F(1, 3763) = 35.20, p < .001) but no significant effect was found for Auto-GEMS (F(1, 3763) = .052, p = .81). Patterns are shown in Supplementary Figure 4.


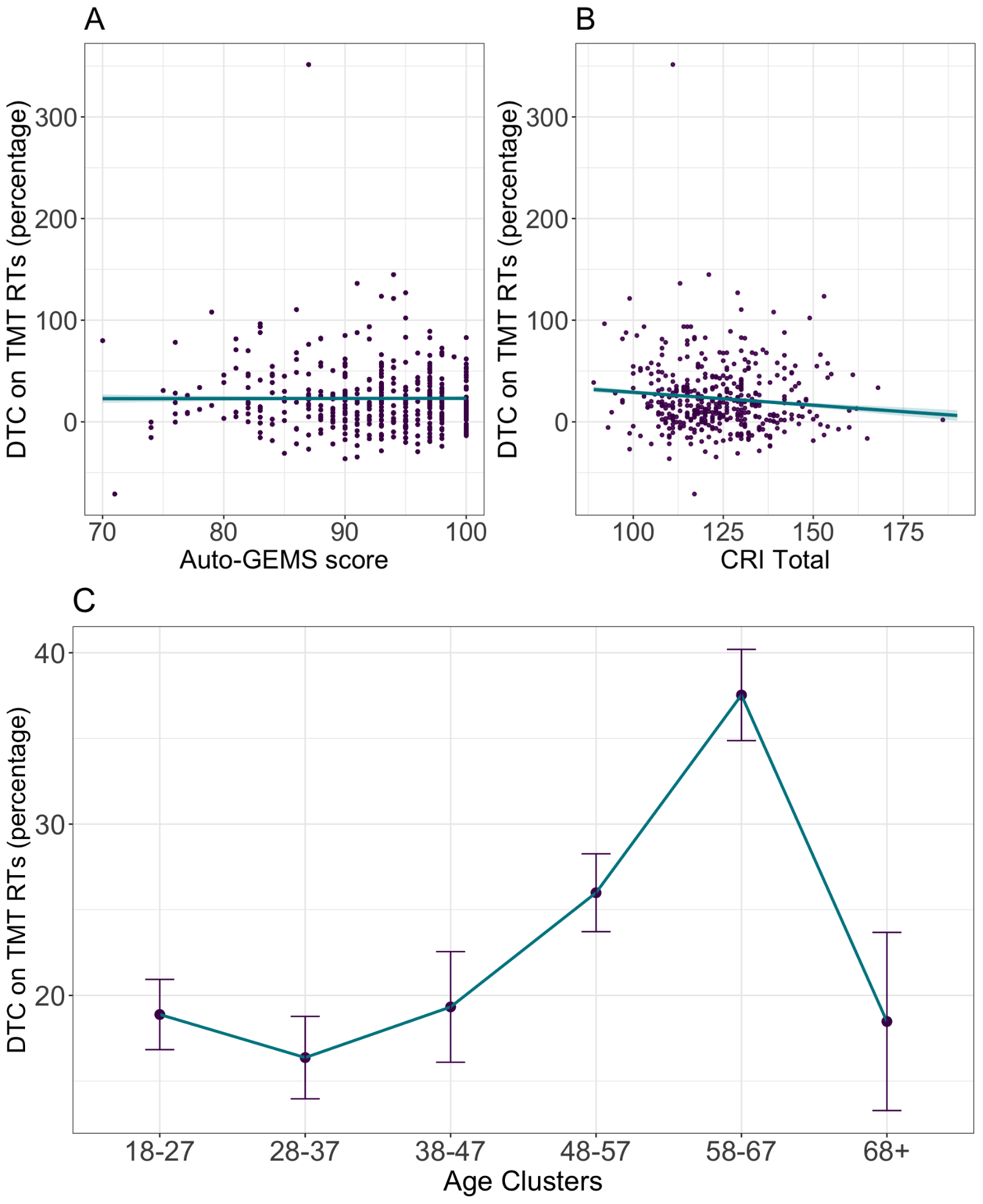


**Supplementary Figure 4.** Predicted DTC (percentage) on TMT RTs is shown as a function of Auto-GEMS score (A), CR (B) and Age Clusters (C). Each point (panel A and B) represents individual DTC. Shaded areas and vertical bars represent the standard error.

**3.2. TAP DTC**

**3.2.1. DTC on TAP Accuracy**

Supplementary Figure 5 show the linear regression between DTC on TAP Accuracy and Auto-GEMS. As discussed in the main text it is only significant predictor of DTC on Accuracy for the TAP.

**
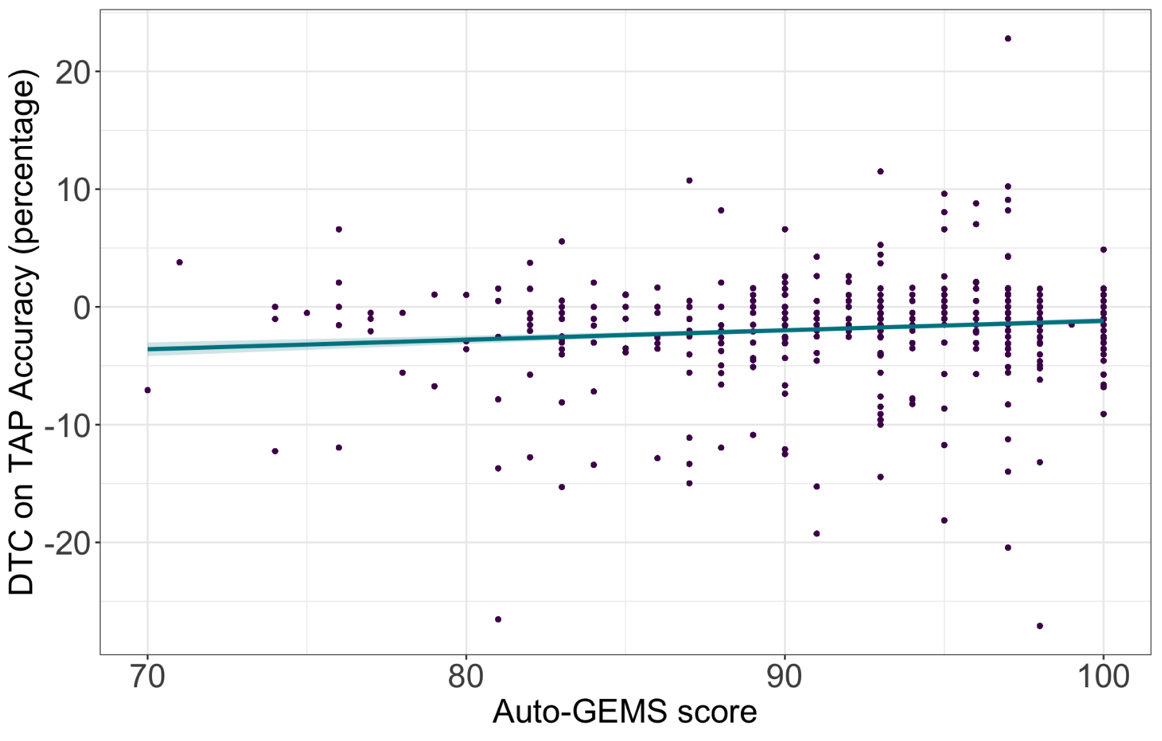
**

**Supplementary Figure 5** Predicted DTC on Accuracy (TAP) as a function of Auto-GEMS score. Each point represents individual Accuracy. Shaded areas and vertical bars represent the standard error. More negative values indicate higher DTC.

**3.2.2. DTC on TAP RTs**

Type II ANOVA on the LM for *RTs DTC* for visual tasks was significantly influenced by *Age* (F(5, 3790) = 15.92, p < .001), and *CR* (F(1, 3790) = 5.67, p = .02) but no significant effect was observed for *Auto-GEMS* (F(1, 3790) = .022, p = .65). Same analysis on *RTs DTC* for auditory task showed significant effects of *Age* (F(5, 3790) = 10.57, p < .001), *CR* (F(1, 3790) = 15.01, p < .001) and *Auto-GEMS* (F(1, 3790) = 38.72, p < .001). Patterns are shown in Supplementary Figure 6 and 7.


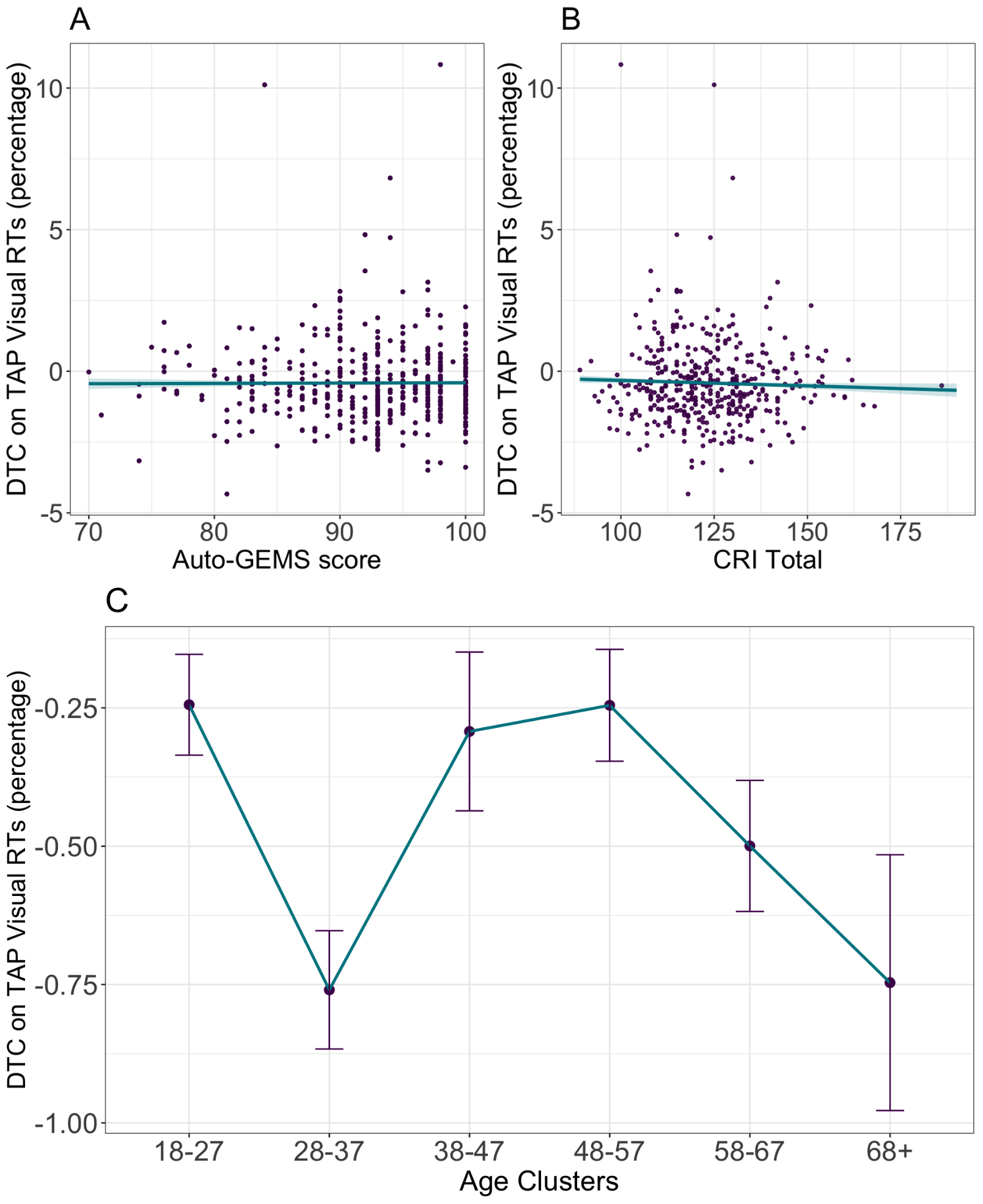


**Supplementary Figure 6.** Predicted DTC (percentage) on RTs of visual task is shown as a function of Auto-GEMS score (A), CR (B) and Age Clusters (C). Each point (panel A and B) represents individual DTC. Shaded areas and vertical bars represent the standard error.

**
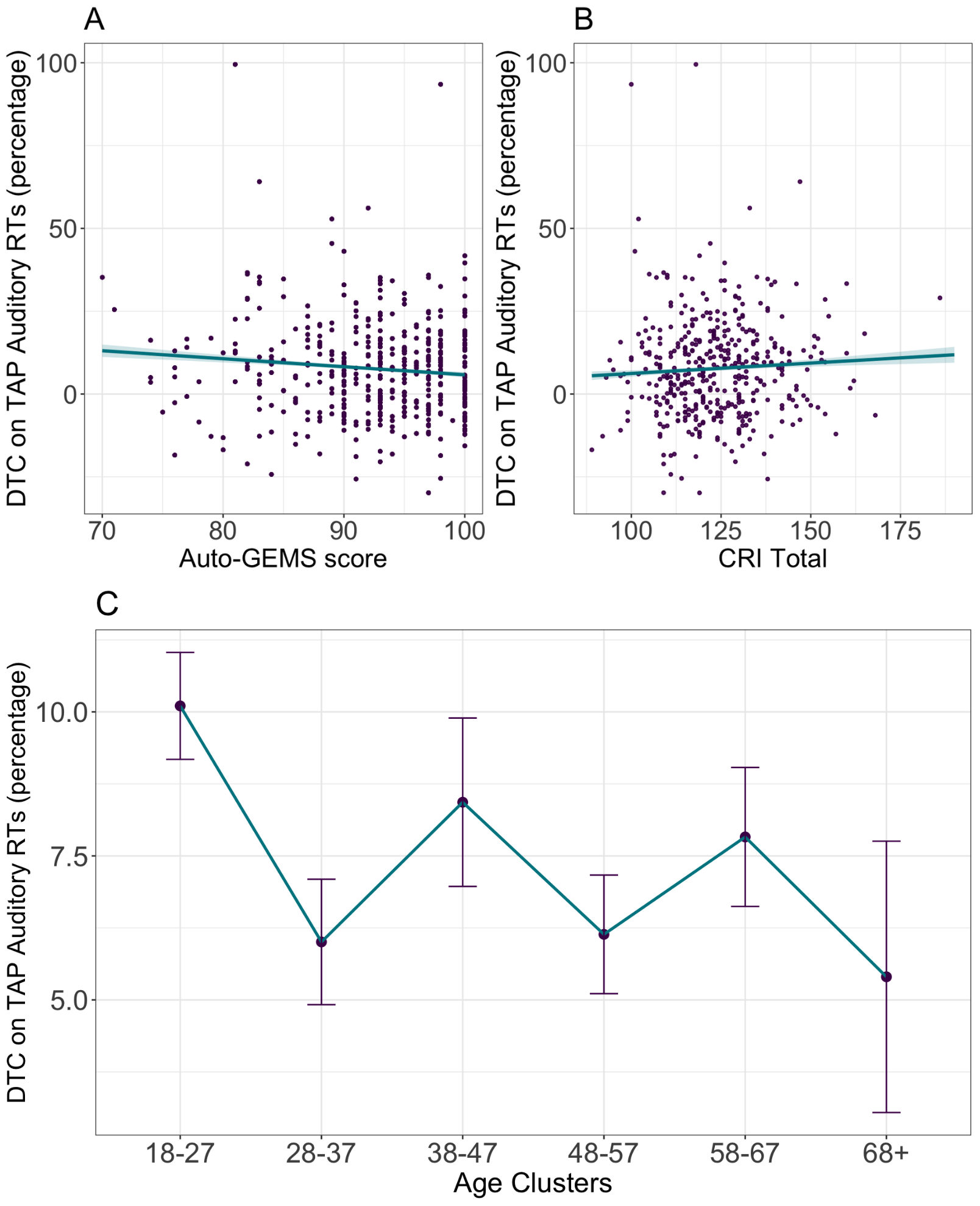
**

**Supplementary Figure 7.** Predicted DTC (percentage) on RTs of auditory task is shown as a function of Auto-GEMS score (A), CR (B) and Age Clusters (C). Each point (panel A and B) represents individual DTC. Shaded areas and vertical bars represent the standard error.

**3.3. MEMO DTC on RTs**

In the context of a Type II ANOVA on the linear model for DTC on RTs, significant effects were observed for *Age* (F(5, 3790) = 8.83, p < .001) *CR* (F(1, 3790) = 56.24 , p < .001) and of Auto-GEMS (F(1, 3790) = 16.97, p < .001). Patterns are shown in Supplementary Figure 7.


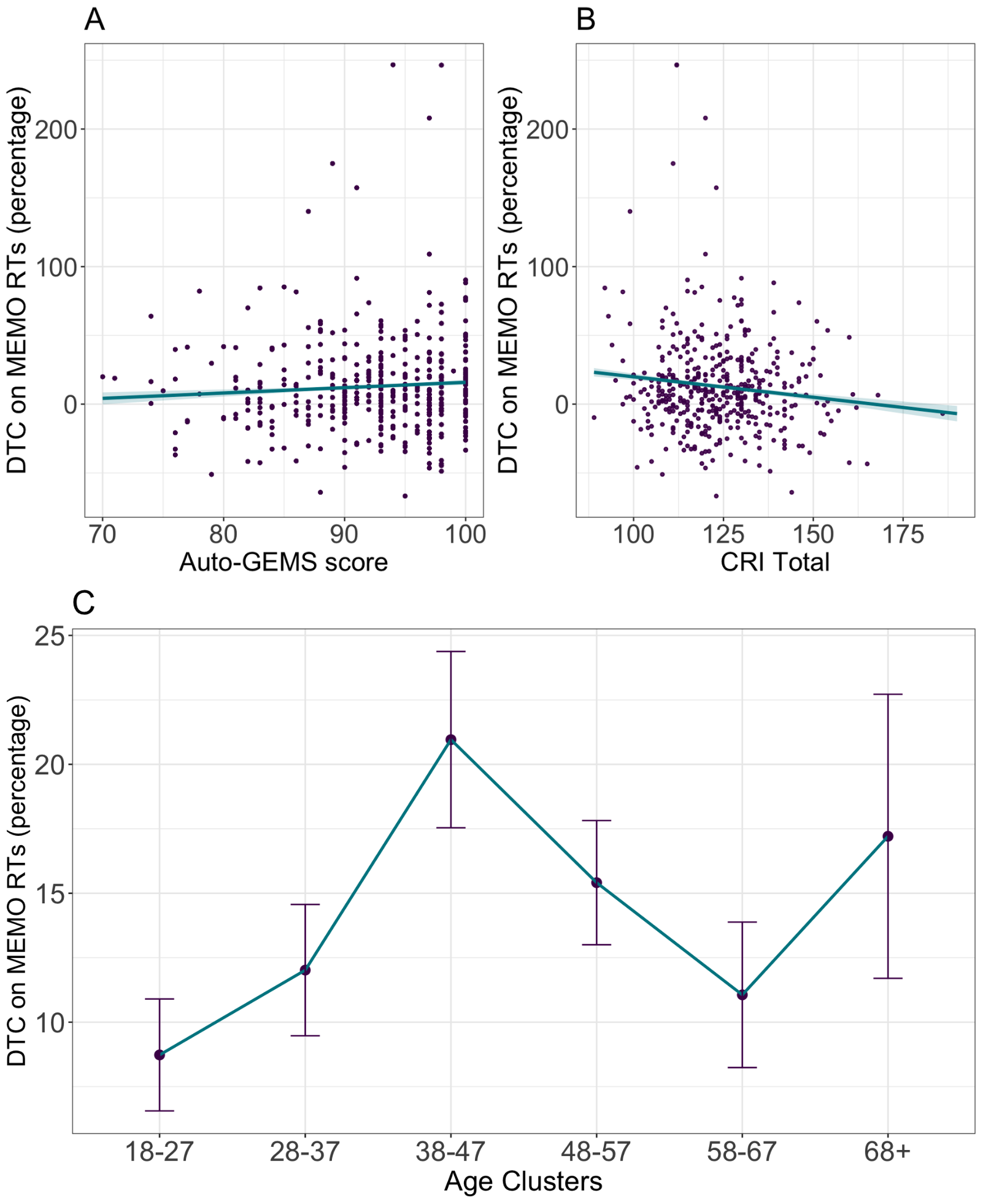


**Supplementary Figure 8.** Predicted DTC (percentage) on RTs of the primary image recognition task is shown as a function of Auto-GEMS score (A), CR (B) and Age Clusters (C). Each point represents individual log-transformed RTs (panel A and B). Shaded areas and vertical bars represent the standard error.

### **4. Correlation Analysis: DTCs on Accuracy/EI and RTs between and within tasks.**

Supplementary Figure 8 shows the correlation of DTC on accuracy/EI between tasks corrected for Age, Auto-GEMS, and CR scores discussed in the main text.


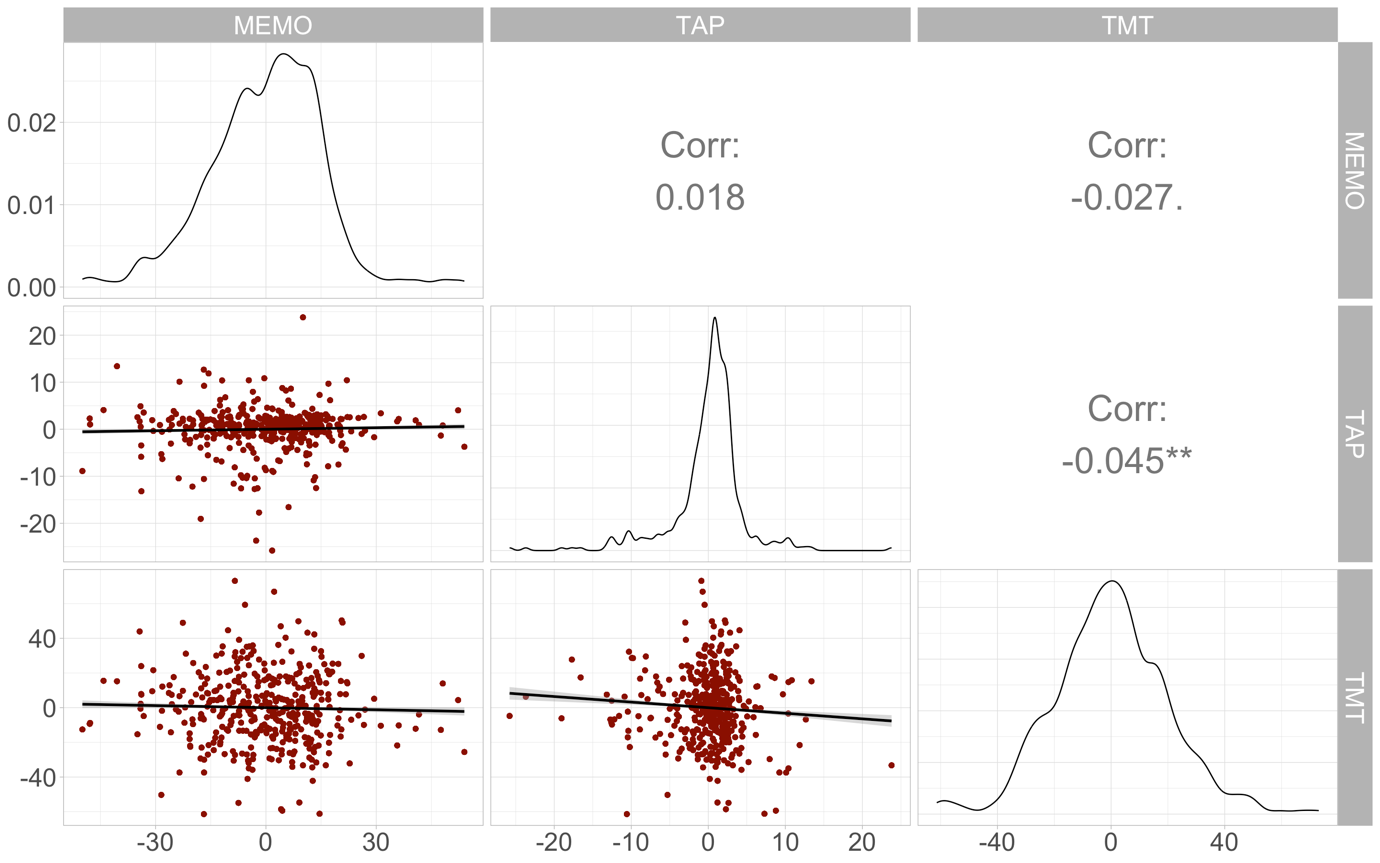


**Supplementary Figure 9.** Spearman’s correlation coefficients (Corr) for the residuals of linear regression models on DTC Accuracy/EI across all tasks. The upper section (above the diagonal) shows the correlation coefficients, while the lower section (below the diagonal) includes scatter plots. Density plots are displayed along the diagonal. ** = p-value < 01.

Similarly correlations between the raw DTCs both Accuracy/EI and RTs across tasks were generally weak (Supplementary Figure 9). Even without controlling for Age, Auto-GEMS, or CR scores, the inter-task correlations remained negligible, further supporting the idea of independent processes underlying different tasks. For instance, inter-task correlations (e.g. MEMO Accuracy - TAP visual RTs : r_spearman_ = .033, p = .15; TAP Accuracy - TMT RTs: r_spearman_ = -.01, p = .38) were either statistically non-significant or of such low magnitude as to be practically negligible.

In contrast, a few intra-task correlations, such as MEMO RTs and MEMO Accuracy (r_spearman_ = .14, p < .001), showed slightly stronger associations, though still weak. These findings highlight the independence of dual-task cost measures across tasks, with minimal shared variance even when covariates are not controlled. The pattern of weak correlations across tasks suggests that significant p-values observed for some comparisons may be attributable to the large sample size rather than meaningful associations between measures.


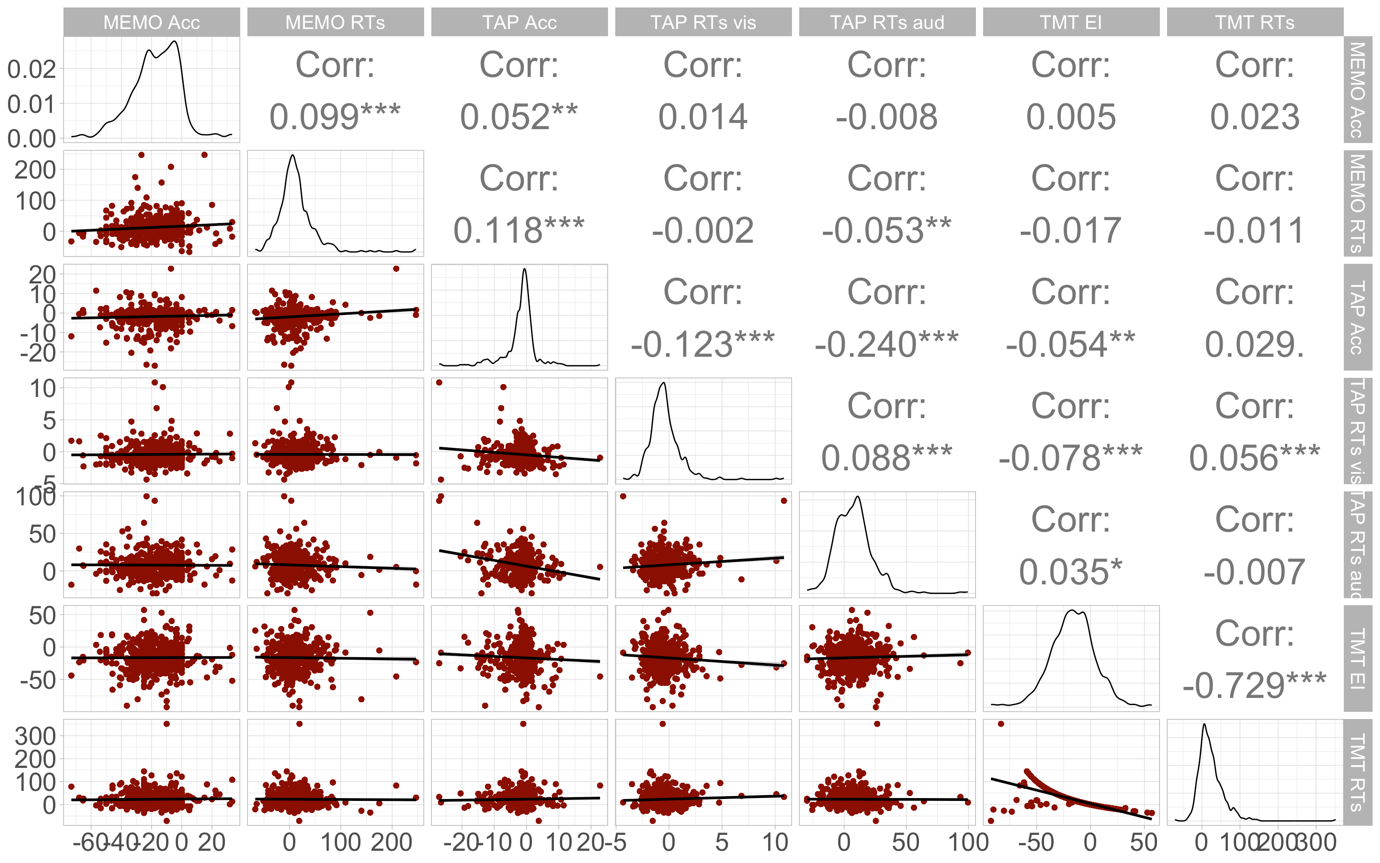


**Supplementary Figure 10.** Spearman’s correlation coefficients for the DTCs Accuracy/EI and RTs within and between tasks. The upper section (above the diagonal) shows the correlation coefficients (Corr), while the lower section (below the diagonal) includes scatter plots. Density plots are displayed along the diagonal. *, **, *** = *p*-value < .05, .01 and .001 respectively**.**

**5. Mediation Analysis for DTC**

The first mediation analysis examined how Auto-GEMS score mediates the relationship between age and DTCs across various tasks (Supplementary Table 8).

Auto-GEMS significantly mediated the relationship between age and DTC on MEMO Accuracy for the image recognition task (indirect effect = -0.0437, p < .001), with 35.55% of the total effect of age on DTC explained by Auto-GEMS. The direct effect of age was also significant and negative (estimate = -.079, p < .001).

Auto-GEMS also significantly mediated the relationship between Age and the DTC for TMT EI (indirect effect = -.036, p < .001). The total effect of age on DTC was significant (estimate = -0.2586, p < .001), with 13.73% of this relationship mediated DTCs on RTs for MEMO and TMT did not show significant mediation effect, confirming that the cognitive efficiency did not influence DTCs on RTs. Results for the DTCs on TAP outcomes are likely biased by the fact that Age did not have a significant direct effect on DTCs for the accuracy and visual RTs. In contrast, a significant direct effect of Age on the DTC for the auditory task RTs was found (direct effect = -0.0633, p = 0.002). However, a substantial portion of this effect is explained by the Auto-GEMS, suggesting that the relationship between Age and DTCs in this case is largely mediated by cognitive efficiency.

**Supplementary Table S8.** Mediation Analysis of Auto-GEMS in the Relationship Between Age and Dual Task Cost (DTC) Across Various Cognitive Tasks

| **Model** | **Metric** | **Estimate** | **95% CI Lower** | **95% CI Upper** | **p-value** |
| --- | --- | --- | --- | --- | --- |
| DTC on MEMO img acc | ACME (Indirect) | -0.0437 | -0.0564 | -0.03 | < .001 *** |
|  | ADE (Direct) | -0.0792 | -0.1116 | -0.05 | < .001 *** |
|  | Total Effect | -0.1229 | -0.1558 | -0.09 | < .001 *** |
|  | **Prop. Mediated** | **0.3555** | 0.2463 | 0.51 | < .001 *** |
| DTC on MEMO img RTs | ACME (Indirect) | -0.0399 | -0.0607 | -0.02 | < .001 *** |
|  | ADE (Direct) | 0.1054 | 0.0333 | 0.17 | 0.004 ** |
|  | Total Effect | 0.0655 | -0.0064 | 0.14 | 0.074 |
|  | Prop. Mediated | -0.6089 | -4.8467 | 2.15 | 0.074 |
| DTC on TMT EI | ACME (Indirect) | -0.0355 | -0.0500 | -0.02 | < .001 *** |
|  | ADE (Direct) | -0.2231 | -0.2696 | -0.18 | < .001 *** |
|  | Total Effect | -0.2586 | -0.3047 | -0.21 | < .001 *** |
|  | **Prop. Mediated** | **0.1373** | 0.0799 | 0.20 | < .001 *** |
| DTC on TMT RTs | ACME (Indirect) | 0.0141 | -0.0085 | 0.04 | 0.19 |
|  | ADE (Direct) | 0.3036 | 0.2363 | 0.38 | < .001 *** |
|  | Total Effect | 0.3177 | 0.2490 | 0.40 | < .001 *** |
|  | Prop. Mediated | 0.0443 | -0.0276 | 0.12 | 0.19 |
| DTC on TAP acc | ACME (Indirect) | -0.0099 | -0.0135 | -0.01 | < .001 *** |
|  | ADE (Direct) | -0.0012 | -0.0119 | 0.01 | 0.81 |
|  | Total Effect | -0.0112 | -0.0199 | 0.00 | 0.01 ** |
|  | **Prop. Mediated** | **0.8886** | 0.3562 | 3.53 | 0.01 ** |
| DTC on TAP RTs vis | ACME (Indirect) | 0.0001 | -0.0009 | 0.00 | 0.84 |
|  | ADE (Direct) | -0.0022 | -0.0054 | 0.00 | 0.22 |
|  | Total Effect | -0.0020 | -0.0047 | 0.00 | 0.17 |
|  | Prop. Mediated | -0.0619 | -1.8603 | 1.40 | 0.75 |
| DTC on TAP RTs aud | ACME (Indirect) | 0.0301 | 0.0193 | 0.04 | < .001 *** |
|  | ADE (Direct) | -0.0633 | -0.0991 | -0.03 | 0.002 ** |
|  | Total Effect | -0.0333 | -0.0645 | 0.00 | 0.036 * |
|  | **Prop. Mediated** | **-0.9046** | -4.3230 | -0.35 | 0.036 * |

**Note:** ACME: Average Causal Mediation Effect; ADE: Average Direct Effect; Prop.: Proportion; RTs: Reaction Times; img: image; vis: visual; aud: auditory; acc: accuracy.; *, **, *** = *p*-value < .05, .01 and .001 respectively**.**

The second mediation analysis explored the role of CR in mediating the relationship between Age and DTC across all tasks (Supplementary Table 9).

The indirect effect of CR on the relationship between Age and DTC for MEMO accuracy on the image recognition task, was positive and significant (indirect effect = 0.0032, p = .01), with 2.61% of the total effect mediated by CR. The direct effect of Age on DTC was significant and negative (estimate = -0.1261, p < .001), confirming the negative relationship between age and DTC. The total effect was also significant and negative (estimate = -0.1229, p < .001).

CR significantly mediated the relationship between age and DTC for the TMT EI (indirect effect = 0.0061, p = .01), with 2.37% of the total effect mediated by CR. The direct effect of age on DTC was negative and significant (estimate = -0.2647, p < .001), and the total effect was also negative and significant (estimate = -0.2586, p < .001). The indirect effect of CR was negative and significant (indirect effect = -0.0078, p = .01), while the direct effect of age was positive and significant (estimate = 0.3254, p < .001). The total effect was positive and significant (estimate = 0.3177, p < .001), and 2.44% of the total effect was mediated by CR.

CR significantly mediated the relationship between age and DTC on RTs in the TAP auditory task (indirect effect = 0.0023, p = .014), with the total effect being negative and significant (estimate = -0.0333, p = .032). The proportion of the effect mediated by CR was significant (estimate = -0.0687, p = .046).

**Supplementary Table S9.** Mediation Analysis of CR in the Relationship Between Age and Dual Task Cost (DTC) Across Various Cognitive Tasks

| Model | Metric | Estimate | 95% CI Lower | 95% CI Upper | p-value |
| --- | --- | --- | --- | --- | --- |
| DTC on MEMO img acc | ACME (Indirect) | .0032 | .0006 | .0100 | .01 ** |
|  | ADE (Direct) | -.1261 | -.1583 | -.1000 | < .001 *** |
|  | Total Effect | -.1229 | -.1548 | -.0900 | < .001 *** |
|  | **Prop. Mediated** | **-.0261** | **-.0627** | **.0000** | **.01 **** |
| DTC on MEMO img RTs | ACME (Indirect) | -.0120 | -.0222 | .0000 | .012 * |
|  | ADE (Direct) | .0775 | .0073 | .1400 | .030 * |
|  | Total Effect | .0655 | -.0090 | .1300 | .082 |
|  | Prop. Mediated | -.1826 | -1.0218 | 1.0600 | .094 |
| DTC on TMT EI | ACME (Indirect) | .0061 | .0013 | .01 | .01 ** |
|  | ADE (Direct) | -.2647 | -.3101 | -.22 | < .001 *** |
|  | Total Effect | -.2586 | -.3029 | -.21 | < .001 *** |
|  | **Prop. Mediated** | **-.0237** | **-.0467** | **.00** | **.01 **** |
| DTC on TMT RTs | ACME (Indirect) | -.0078 | -.0156 | .00 | .01 ** |
|  | ADE (Direct) | .3254 | .2558 | .4000 | < .001 *** |
|  | Total Effect | .3177 | .2492 | .3900 | < .001 *** |
|  | **Prop. Mediated** | **-.0244** | **-.0516** | **.0000** | **.01 **** |
| DTC on TAP acc | ACME (Indirect) | -.0000 | -.0006 | .0000 | .976 |
|  | ADE (Direct) | -.0112 | -.0192 | -.0100 | .012 * |
|  | Total Effect | -.0112 | -.0199 | -.0100 | .014 * |
|  | Prop. Mediated | .0004 | -.0599 | .0800 | .978 |
| DTC on TAP RTs vis | ACME (Indirect) | -.0001 | -.0003 | .0000 | .058 |
|  | ADE (Direct) | -.0019 | -.0051 | .0000 | .196 |
|  | Total Effect | -.0020 | -.0052 | .0000 | .166 |
|  | Prop. Mediated | .0694 | -.6524 | .6200 | .216 |
| DTC on TAP RTs aud | ACME (Indirect) | .0023 | .0003 | .0000 | .014 * |
|  | ADE (Direct) | -.0355 | -.0648 | -.0100 | .030 * |
|  | Total Effect | -.0333 | -.0645 | .0000 | .032 * |
|  | **Prop. Mediated** | **-.0687** | **-.4004** | **.0000** | **.046 *** |

**Note:** ACME: Average Causal Mediation Effect; ADE: Average Direct Effect; Prop.: Proportion; RTs: Reaction Times; img: image; vis: visual; aud: auditory; acc: accuracy.; *, **, *** = *p*-value < .05, .01 and .001 respectively**.**
